# Exploring the Growth Dynamics of Size‐Selected Carbon Atomic Wires with In Situ UV Resonance Raman Spectroscopy

**DOI:** 10.1002/smll.202403054

**Published:** 2024-07-28

**Authors:** Pietro Marabotti, Sonia Peggiani, Simone Melesi, Barbara Rossi, Alessandro Gessini, Andrea Li Bassi, Valeria Russo, Carlo Spartaco Casari

**Affiliations:** ^1^ Department of Energy Micro and Nanostructured Materials Laboratory – NanoLab Politecnico di Milano Via Ponzio 34/3 Milano 20133 Italy; ^2^ Institut für Physik Humboldt‐Universität zu Berlin Newtonstraße 15 12489 Berlin Germany; ^3^ Elettra Sincrotrone Trieste S.S. 114 km 163.5 Basovizza Trieste 34149 Italy

**Keywords:** carbon atomic wires, in situ, polyynes synthesis, pulsed laser ablation, size‐dependent, solvent, UV Resonance Raman spectroscopy

## Abstract

Short carbon atomic wires, the prototypes of the lacking carbon allotrope carbyne, represent the fundamental 1D system and the first stage in carbon nanostructure growth, which still exhibits many open points regarding their growth and stability. An in situ UV resonance Raman approach is introduced for real‐time monitoring of the growth of carbon atomic wires during pulsed laser ablation in liquid without perturbing the synthesis environment. Single‐chain species’ growth dynamics are tracked, achieving size selectivity by exploiting the peculiar optoelectronic properties of carbon wires and the tunability of synchrotron radiation. Diverse solvents are systematically explored, finding size‐ and solvent‐dependent production rates linked to the solvent's C/H ratio and carbonization tendency. Carbon atomic wires’ growth dynamics reveal a complex interplay between formation and degradation, leading to an equilibrium. Water, lacking in carbon atoms and reduced polyynes solubility, yields fewer wires with rapid saturation. Organic solvents exhibit enhanced productivity and near‐linear growth, attributed to additional carbon from solvent dissociation and low relative polarity. Exploring the dynamics of the saturation regime provides new insights into advancing carbon atomic wires synthesis via PLAL. Understanding carbon atomic wires’ growth dynamics can contribute to optimizing PLAL processes for nanomaterial synthesis.

## Graphical abstract

Time‐resolved, in situ UV resonance Raman approach to monitor carbon atomic wires growth via pulsed laser ablation in liquid. This approach tracks solvent‐dependent, size‐selected wires formation, correlating their Raman responses with concentration as a function of ablation time. It elucidates the synthesis, formation, and degradation mechanisms of carbon atomic wires via pulsed laser ablation and solvent‐dependent production rates.

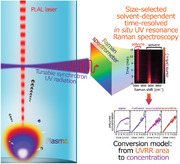

## 1 Introduction

Carbon remarkable ability to assemble in diverse structures has captivated researchers’ interest across many scientific and technological fields, ranging from astronomy to material science and nanotechnology. In astrochemistry, fullerene was first detected in space,^[^ ^1^ ^]^ and the presence of carbon clusters in interstellar dust is pivotal to understanding the formation of large organic molecules, eventually related to the origin of life.^[^ ^2^, ^3^ ^]^ Short linear sp‐carbon chains or carbon atomic wires have been detected in diffuse interstellar bands and carbon clusters synthesized in a laboratory. Further, they are considered a key step in the formation of larger carbon aggregates, including fullerenes (e.g., the so‐called fullerene road) as the first stages of growth under strong non‐equilibrium conditions, possibly explaining their elusive nature.^[^ ^4^, ^5^, ^6^, ^7^, ^8^, ^9^, ^10^ ^]^

Carbon atomic wires are among the most intriguing materials currently under investigation. These linear systems are the finite realization of carbyne, the ideal 1D linear chain made of sp‐hybridized carbon atoms.^[^ ^11^, ^12^, ^13^ ^]^ The interest in these systems arises from the outstanding properties predicted for carbyne, ranging from superior optical absorption and thermal transport to the transition between a metallic phase (i.e., cumulene) and a semiconducting one (i.e., polyyne).^[^ ^14^ ^]^ Confined carbyne, i.e., long (>1000 carbon atoms) linear carbon chains encapsulated in double‐walled carbon nanotubes, represents the closest approach to carbyne.^[^ ^12^, ^15^ ^]^ Researchers recently discovered its exceptional optical properties, like its paramount resonance Raman cross section, higher than any other known material.^[^ ^14^, ^16^, ^17^, ^18^ ^]^ In this framework, carbon atomic wires represent small carbyne‐like chains with less than 100 sp‐hybridized carbon atoms and end‐capped at both edges by a large set of possible functional groups.^[^ ^11^, ^12^, ^13^ ^]^ It is possible to tailor carbon atomic wires’ optical, thermal, mechanical, and electronic properties by varying their length and termination.^[^ ^19^, ^20^, ^21^, ^22^, ^23^, ^24^, ^25^, ^26^, ^27^, ^28^, ^29^, ^30^, ^31^, ^32^ ^]^ However, this requires high control and flexibility in the synthesis process. Chemical synthesis methods provide an appropriate control, producing chains with selected lengths and terminations, but they are not flexible and fast enough to allow a proper scale‐up to an industrial production level.^[^ ^11^, ^20^, ^33^ ^]^ On the other hand, physical synthesis techniques possess these capabilities, but they grant only limited control over the synthesis process and final products so far.^[^ ^11^, ^34^, ^35^ ^]^

Among the physical techniques, pulsed laser ablation in liquid (PLAL) is the most versatile, flexible, and simple method to synthesize a wide range of carbon atomic wires.^[^ ^36^ ^]^ It employs a short laser pulse, from the *fs* to the *ns* range, to irradiate a solid target or a powder immersed in a liquid medium or focus the pulse within the solvent. This technique allows to obtain chains with different sizes and terminations by selecting the solvent,^[^ ^37^, ^38^, ^39^, ^40^, ^41^, ^42^, ^43^, ^44^, ^45^, ^46^, ^47^, ^48^, ^49^, ^50^ ^]^ target,^[^ ^34^, ^38^, ^51^, ^52^, ^53^, ^54^, ^55^, ^56^, ^57^ ^]^ pulse duration,^[^ ^42^, ^58^, ^59^, ^60^, ^61^, ^62^, ^63^, ^64^ ^]^ laser wavelength,^[^ ^34^, ^51^, ^65^, ^66^ ^]^ and energy of the laser beam^[^ ^34^, ^36^, ^42^ ^]^ properly. Even if many works report carbon atomic wires’ synthesis via PLAL, many aspects governing the formation process remain elusive.^[^ ^36^, ^51^ ^]^ The linear chains’ growth is intricately influenced by the interplay between two competing phenomena: polymerization reactions, driving chain elongation, and hydrogenation reactions, which result in chain termination, typically with hydrogen atoms.^[^ ^36^, ^51^ ^]^ This synthesis process is inherently based on the interaction of radical species, and the polymerization is believed to occur through adding carbon dimers and/or ethynyl radicals.^[^ ^36^, ^48^, ^51^ ^]^ Unfortunately, direct proof of these processes during ablation remains challenging due to their ultrafast timescales (ranging from *fs* to hundreds of *ns* ^[^ ^36^, ^67^, ^68^, ^69^ ^]^) and the complex environment of PLAL experiments. In this context, in situ (i.e., during the growth) experiments hold promise for deepening our understanding even though a characterization technique with high sensitivity to the local chemical bond is required to monitor the formation process.

Raman spectroscopy provides a powerful characterization technique for carbon atomic wires and a non‐disruptive, contactless, and fast method to monitor them and eventually develop an in situ diagnostic tool. Indeed, carbon atomic wires possess a remarkable Raman response, which consists of a fingerprinting Raman‐active vibration called ECC (from the Effective Conjugation Coordinate theory) or α mode.^[^ ^70^, ^71^ ^]^ The α mode ranges from 1800 to 2300 cm^−1^, in a spectral region where other carbon allotropes, like byproducts simultaneously produced with carbon atomic wires during the ablation, do not exhibit any Raman‐active mode.^[^ ^71^ ^]^ Its frequency modulates with the chain length and termination, providing a unique Raman signal for each species and allowing us to track single‐chain species dynamics.^[^ ^71^ ^]^ Depending on the chain length and terminations, other Raman‐active vibrations are present in the characteristic frequency range of the α mode, like, for example, the β mode of hydrogen‐capped polyynes^[^ ^25^, ^70^, ^72^ ^]^ or the CN stretching mode of cyano‐capped polyynes.^[^ ^72^ ^]^

Unfortunately, the typical concentration of carbon atomic wires in mixtures produced by PLAL is well below the threshold (≈10^−3^ mol L^−1^) to collect good‐quality Raman spectra, and some signal enhancer is required. In our previous work, we conducted an in situ experimental campaign using a surface‐enhanced Raman scattering (SERS) probe.^[^ ^73^ ^]^ Our findings revealed an intriguing aspect: carbon atomic wires degrade already during their synthesis, influencing the final yield and chains’ size distribution. However, our SERS data exhibit the convolution of SERS signals from wires of different lengths and terminations and are mediated by the interaction between carbon atomic wires and metal nanoparticles. Hence, gaining insights into the growth dynamics of size‐ and termination‐selected wires remains challenging.

We recently showed how Resonance Raman spectroscopy resulted in an outstanding characterization method for carbon atomic wires and, in particular, polyynes. Indeed, by finely tuning the excitation wavelengths to the electronic transitions of carbon atomic wires, their Raman signal can be significantly enhanced, making low concentrated samples, down to 10^−8^ mol L^−1^, observable.^[^ ^25^, ^72^ ^]^ Moreover, polyynes possess intense and sharp electronic transition in the UV range (from 198 to 400 nm), whose energies modulate with the chain length and terminations,^[^ ^11^, ^37^, ^45^, ^74^ ^]^ providing the required selectivity to follow the dynamics of the single chain in a polydisperse mixture, i.e., observing the resonance Raman signal of wires selected by length and termination in a polydispersed solution obtained through PLAL. The increment of the detection limit due to the resonance enhancement allows us to collect Raman spectra in a reasonable time interval (i.e., a few seconds) compared to the characteristic ablation times (i.e., several minutes). Based on these results, Resonance Raman spectroscopy is the candidate technique for real time in situ monitoring of the growth of carbon atomic wires during their synthesis by laser ablation in liquid with size‐selected resolution.

In this work, we designed and set up an in situ multi‐wavelength UV Resonance Raman system to monitor the *real time* growth of carbon atomic wires through our PLAL apparatus. The fine tunability required to match the resonance condition with each different wire (i.e., step of ≈1 nm in the 200–272 nm range) is provided using the synchrotron radiation as Raman excitation source. We perform ablation in water and organic solvents (methanol, isopropanol, and acetonitrile), selected for their UV transparency, stability under laser ablation, and varying polarity and C/H ratio. This selection allows us to explore how these key parameters influence polyyne formation yield and stability. Instead, water was included as a negative control to highlight the role of organic solvents in providing additional carbon sources. We track the behavior of four size‐selected hydrogen‐capped polyynes, i.e., HC_8_H, HC_10_H, HC_12_H, and HC_14_H. We develop an effective, simple, and analytical method to correct the raw Raman data from self‐absorption induced by the remarkable absorption of the ablation mixture at the Raman excitation wavelength and extract the concentration of each wire as a function of the ablation time. We detect the onset of degradation processes simultaneously with the formation of carbon atomic wires, eventually leading to an equilibrium between formation and degradation. Observing the dynamics of carbon atomic wires’ growth, we evaluate the production rate during ablation and other relevant parameters, like the maximum concentration reachable for each chain (i.e., at the formation‐degradation equilibrium) and the time needed to reach that concentration. Our findings provide further insight into carbon atomic wires’ growth dynamics and carbon nanostructures in strong out‐of‐equilibrium conditions and the effect of the liquid environment on the growth of size‐selected chains.

## 2 Results and Discussion

**Figure 1:**
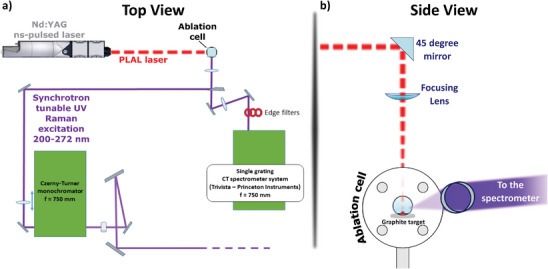
Pulsed laser ablation setup integrated with UV Raman system. a) Top and b) side views of the pulsed laser ablation setup integrated with the synchrotron‐based UV Resonance Raman system of the IUVS beamline to perform in situ monitoring of the growth of polyynes.

We in situ monitored the growth of carbon atomic wires (i.e., polyynes) during pulsed laser ablation in liquid (PLAL) experiments by integrating our PLAL system with the multi‐wavelength, synchrotron‐based UV Raman scattering setup of the IUVS beamline (Elettra Sincrotrone Trieste, Italy), as illustrated in **Figure** **1**. After optimizing the signal, we set an acquisition time of 10 s per spectrum, which is fast enough to provide reliable statistics for the whole growth dynamics (15 min) and adequately long to obtain a good signal‐to‐noise ratio during the early stages of ablation.

The exceptional selectivity of UV Resonance Raman (UVRR) allowed us to track the evolution of single‐chain species. As a proof of concept, we collected two UVRR spectra of polydispersed mixtures containing different carbon atomic wires and byproducts. After tuning the synchrotron radiation to a selected wavelength in resonance with the most intense vibronic transition of a specific polyyne, the UVRR spectra of these mixtures predominantly exhibit Raman features (the α and β modes) coming from the selected polyynes (i.e., HC_8_H and HC_10_H), as shown in Figure S1 (Supporting Information).

To collect UVRR spectra, we focused the synchrotron‐based UV excitation ≈7 mm above the graphite target surface. This geometry allowed us to avoid any interference with UVRR measurement coming from the plasma plume since the length of the plasma plume in our conditions is well below 3 mm, as observed in ablations at much higher fluences (tens of J cm^−2^)^[^ ^69^, ^75^, ^76^, ^77^, ^78^ ^]^ compared to our experiments. Moreover, in this way, we prevented any delay time issue since the travel time from the growth region to the focal point is of the order of a few *µs* since polyynes are ejected from the nucleation site at supersonic speed (≈1500 m s^−1^),^[^ ^73^, ^75^, ^78^, ^79^ ^]^ carried by shockwaves generated after the collapse of the plasma plume.

To select suitable solvents for ablation experiments, we considered several factors critical to our study. First, the UV transparency of the solvents within the beamline wavelength range of interest was vital, as it directly impacts the efficiency and accuracy of UVRR spectroscopy. Using UV‐transparent solvents ensures the required penetration in the liquid environment of the UV Raman excitation and prevents any solvent‐related absorption effects. Additionally, we aimed to explore how the solvents’ polarity and C/H ratio influence polyynes’ formation, dynamics, and stability during their synthesis by PLAL. Indeed, previous studies showed that solvents with reduced polarity showed enhanced polyynes’ production with PLAL,^[^ ^37^, ^38^, ^80^, ^81^ ^]^ while solvents with a high C/H ratio promote the polymerization and the formation of longer chains.^[^ ^36^, ^37^, ^45^, ^81^, ^82^ ^]^ For these reasons, we chose water, methanol, isopropanol, and acetonitrile. Water, with a UV cutoff at 190 nm, serves as a highly polar environment (relative polarity 1, as listed in Refs. [83, 84]) and acts as a negative control to emphasize the role of organic solvents as additional carbon sources for the polymerization reaction of polyynes. Methanol, with a UV cutoff at 205 nm, is a slightly less polar solvent than water, with a relative polarity of 0.762^[^ ^83^, ^84^ ^]^ and a C/H ratio of 0.33. Isopropanol, with a UV cutoff at 205 nm, has a lower polarity (0.564^[^ ^83^, ^84^ ^]^) and a higher C/H ratio (0.5). Acetonitrile, with a UV cutoff at 190 nm, presents the lowest polarity among the selected solvents (0.460^[^ ^83^, ^84^ ^]^) and the highest C/H ratio (0.67). These solvents cover a broad spectrum of chemical properties, allowing us to systematically study solvent effects on polyynes’ growth dynamics.

**Figure 2:**
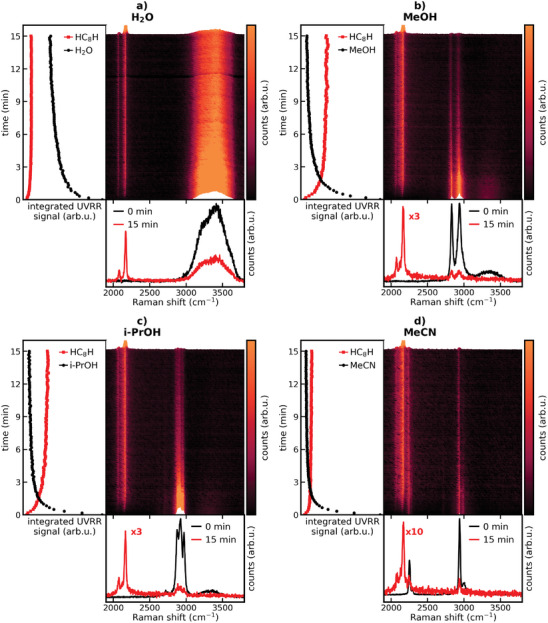
In situ UVRR spectra collected at 226 nm as excitation wavelength in a) water, b) methanol (MeOH), c) isopropanol (i‐ProH), and d) acetonitrile (MeCN) during 15 min of ablation (1064 nm ablation laser, 15 min of ablation time, 50 mJ per pulse), in the colormap of each panel. The integrated UVRR Raman signals of HC_8_H's α mode (red squares) and the relevant solvent Raman band (black circles, see main text) are displayed in the left‐hand box of each panel. Fit errors (see Section S1, Supporting Information) are shown with error bars. The first (0 min) and last (15 min) spectra are reported in the bottom box of each panel. The 15 min spectra are multiplied by a variable factor to improve the visualization.

**Figure** **2** shows the evolution of the in situ UVRR signals collected at 226 nm as excitation wavelength during ablation experiments in different solvents (water in panel a, methanol in panel b, isopropanol in panel c, and acetonitrile in panel d). We obtained analogous in situ UVRR dynamics at 251, 272, and 264 nm, shown in Figures S2–S4 (Supporting Information), respectively. We chose these wavelengths since they are in resonance with the most intense peak (0‐0 vibronic absorption) of hydrogen‐capped polyynes with 8, 10, and 12 sp‐carbon atoms, i.e., HC_8_H (226 nm), HC_10_H (251 nm), and HC_12_H (272 nm), as shown in Figure S5 and Table S1 (Supporting Information). We excited the hydrogen‐capped polyyne with 14 sp‐carbon atoms (HC_14_H) at its 0–2 vibronic peak (i.e., 264 nm) since this is the first available transition for this wire (i.e., 0‐0 at 295 nm and 0–1 at 280 nm, see Figure S5, Supporting Information) in the UV excitation range of the beamline (200‐272 nm, see Method section). The ablation parameters selected in all experiments are listed in the Methods section.

UVRR spectra in Figure 2 (Raman excitation at 226 nm) display two main groups of signals at distinct frequency ranges. Below 2200 cm^−1^, we observe two main bands at ≈2175 and 2085 cm^−1^ (averaging across different solvents; see Figure S5b and Table S2 in the Supporting Information), growing as ablation proceeds. Since we are in resonance with the 0‐0 vibronic peak of HC_8_H (Figure S5a, Supporting Information), these signals correspond to its α and β modes (Figure S5b and Table S2, Supporting Information), in agreement with data in Refs. [25, 70] Moreover, the band at 2170 cm^−1^ displays a less intense shoulder at lower frequencies (≈2120 cm^−1^, averaging across different solvents, as displayed in Figure S5b, Supporting Information), visible for ablations in MeOH, i‐PrOH, and MeCN (see Figure 2b–d, respectively) at longer ablation times. We assigned this signal to the α mode of HC_10_H (Table S2, Supporting Information), enhanced by matching its 0‒2 vibronic transition at ≈226 nm (Figure S5a, Supporting Information).^[^ ^25^, ^37^, ^70^, ^72^, ^85^ ^]^ A summary of the results presented herein is available in Table S2 (Supporting Information).

Correspondingly, in situ UVRR spectra collected at 251 nm (see Figure S2, Supporting Information) and 272 nm (see Figure S3, Supporting Information) present features similar to what we observed at 226 nm. Notably, the α modes of HC_10_H (2127 cm^−1^, averaging across different solvents; see Figure S5c,d, Supporting Information) and HC_12_H (2100 cm^−1^, averaging across different solvents; see Figure S5e,f, Supporting Information) emerge as the predominant polyynic Raman peaks in the UVRR spectra recorded at 251 nm and 272 nm, respectively, according to literature data.^[^ ^25^, ^70^, ^72^, ^85^ ^]^ A relatively weak HC_12_H signal was observed in water ablations, indicating reduced productivity of longer polyynes in this medium. Furthermore, we observe the presence of their β modes and weaker signals corresponding to HC_12_H and HC_14_H's α modes in 251 and 272 nm spectra, respectively. Refer to Table S2 in the Supporting Information for a comprehensive overview of these results.

In situ measurements carried out at 264 nm reveal two distinct peaks at 2095 and 2060 cm^−1^, corresponding to HC_12_H and HC_14_H's α modes (averaging across different solvents; see Figure S5g,h in the Supporting Information), consistent with previous studies.^[^ ^25^, ^37^, ^70^, ^72^, ^85^ ^]^ Detailed information is provided in Table S2 in the Supporting Information. In situ UVRR spectra are provided for MeOH and MeCN, while no data is available for water due to the expected lower productivity for HC_14_H compared to HC_12_H, for which we recorded only a weak signal.^[^ ^34^, ^37^, ^51^ ^]^ Moreover, spectra in i‐PrOH are not reported due to the highly noisy background obscuring Raman signals, possibly originating from some plasma plume emissions particularly strong in ablations in i‐PrOH, while they are weaker in ablations in MeOH and MeCN (see spectral disturbances above 3000 cm^−1^ in Figure S4 in the Supporting Information).

From these observations, this innovative approach demonstrates its unique ability to provide direct access to spectroscopic information related to single‐chain species growth despite the presence in the ablated solutions of many different polyynes with different chain lengths and terminations and carbon‐based byproducts like hydrocarbons.^[^ ^34^, ^37^, ^51^, ^65^ ^]^ This selectivity is unfeasible with other in situ techniques, like the surface‐enhanced Raman scattering probe we developed in our previous work.^[^ ^73^ ^]^

Regarding the other groups of signals present in UVRR spectra above 2800 cm^−1^, they are assigned to the CH or OH stretching modes of the different solvents. Water displays its OH stretching band from ≈2900 to 3800 cm^−1^ (see Figure 2a). The same Raman band is visible, even if very weak, in alcohols (see Figure 2b,c) ‒, i.e., methanol and isopropanol. Instead, alcohols (Figure 2b,c) and acetonitrile (Figure 2d) exhibit intense Raman‐active CH stretching modes between 2800 and 3000 cm^−1^. Moreover, acetonitrile possesses a CN stretching vibration centered at ≈2258 cm^−1^.

To correctly evaluate the evolution of the growth of the main species selected by matching their resonant condition, we fit each spectrum with a multi‐curve model (including solvent signals) with a linear baseline correction. Regarding the solvents, we used different Raman bands to track their behaviors: we integrated the OH stretching band for water and the CH stretching modes for methanol, isopropanol, and acetonitrile. The fitting model is described in Section S1 in the Supporting Information. The corresponding integrated UVRR signals (i.e., the ascribed area of the Lorentzian curves) are displayed in the left box of each panel of Figure 2 by exciting at 226 nm ‒ in resonance with the 0‒0 vibronic peak of HC_8_H ‒ and Figures S2–S4 (Supporting Information) by exciting at 251, 272, and 264 nm, respectively ‒ in resonance with the 0‒0 vibronic peak of HC_10_H and HC_12_H and the 0‒2 vibronic peak of HC_14_H, respectively.

The evolution of HC_8_H's α mode and the solvent's Raman bands (see Figure 2) reveals a simultaneous increase in the intensity of polyynes peaks and a decrease in the Raman response of solvent peaks, consistently observed across all solvents. The decrease in solvent Raman intensity is particularly remarkable given that the solvent concentration did not vary during the ablations, and the focus condition remained unperturbed. This counterintuitive trend can be attributed to self‐absorption (SA), a well‐known phenomenon in resonance Raman experiments.^[^ ^86^, ^87^, ^88^ ^]^ Typically, the SA effect reduces a sample's Raman response due to increased absorption at the Raman excitation wavelength. In our study, SA is not attributable to the solvents, as we deliberately selected UV‐transparent solvents within the relevant beamline wavelength range. Instead, it arises from the concurrent production of polyynes and byproducts (like hydrocarbons),^[^ ^36^, ^37^, ^38^, ^39^, ^40^, ^45^, ^47^, ^48^, ^57^, ^65^, ^73^, ^89^ ^]^ which effectively absorb synchrotron‐based Raman excitation. The SA is especially sensitive to polyyne's concentration, as we tuned the Raman excitation in resonance with a vibronic transition of each polyyne. Consequently, polyynes and byproducts absorb a portion of the UV power at the focal point within the mixture, enhancing SA and explaining the solvent's reduced Raman response.

Instead, the apparent saturation of polyynes’ UVRR signal is caused by the raising mixture's SA and increased polyynes’ Raman signal, both stemming from polyynes’ concentration growth. However, ex situ data collected under similar ablation conditions in other studies demonstrated a continuous increase of all the H‐capped polyynes within the first 60 min of ablation in acetonitrile^[^ ^73^ ^]^ and between 15 min (for HC_10_H) and 25 min (for HC_8_H) in water.^[^ ^66^ ^]^ Therefore, we anticipate that in situ UVRR data will exhibit similar behavior, and a correction for SA is necessary to accurately evaluate polyynes’ actual growth dynamics.

Addressing SA must be considered carefully because both the incoming synchrotron beam and the backscattered Raman photons are absorbed by the mixture, as shown by the UV‐Vis absorption spectra of mixtures in Figure S8 (Supporting Information). We can distinguish slightly different absorbances experienced by the backscattered Raman photons of the α mode (from 2000 to 2200 cm^−1^) or solvent Raman bands (CH stretching from 2800 to 3000 cm^−1^ and OH stretching from 2900 to 3800 cm^−1^). This contribution, however, is always weaker compared to the SA at the Raman excitation wavelength. Given the exponential nature of absorption, as a first approximation, we will consider the latter component as predominantly affecting in situ UVRR measurements.

We developed an empirical model to correct in situ UVRR data for SA, using the solvent Raman band as internal reference, as detailed in Section S2 (Supporting Information). As discussed earlier, the area of the Raman band of each solvent ($A_{s}\left(t\right)$, where t is the ablation time) should remain equal to its initial value ($A_{s}(0)$). By multiplying this factor with the area of polyyne's α mode ($A_{p}(t)$), we obtain the SA‐corrected integrated polyynes’ signal ($A_{p}^{\prime }(t)$):

(1) $$A_{p}^{\prime }\;\left(t\right)=A_{p}\;\left(t\right)\frac{A_{s}\left(0\right)}{A_{s}\left(t\right)}$$

The use of this empirical approach is justified by the unfeasibility to meet the requirements of analytical expression, as the model reported in Ref. [86] in terms of knowledge of physical aspects of polyynes and byproducts and geometrical details of the setup (see Section S2, Supporting Information).

We can further analyze the corrected UVRR integrated polyynes’ signal to obtain more significant information about the growth. Specifically, using ex situ UVRR measurements of size‐selected H‐capped polyynes with known concentration, we established a linear relationship between the integrated signal and concentration (see Section S3, Supporting Information). This model links the integrated UVRR signal (${A^{\prime }}_{p,\;\lambda }\left(t\right)$) of each specific chain (p) collected at the characteristic excitation wavelength (λ) and ablation time (t) to its corresponding concentration ($c_{p,\lambda }\left(t\right)$) through this equation:

(2) $$c_{p,\lambda }\left(t\right)=A_{p,\lambda }^{\prime }\left(t\right)\cdot k_{\mathrm{UVRR}\to c}\left(p,\lambda \right)$$

where we determined a conversion factor, $k_{\mathrm{UVRR}\to c}\left(p,\lambda \right)$, from the calibration curve of each polyyne at each specific wavelength.

**Figure 3:**
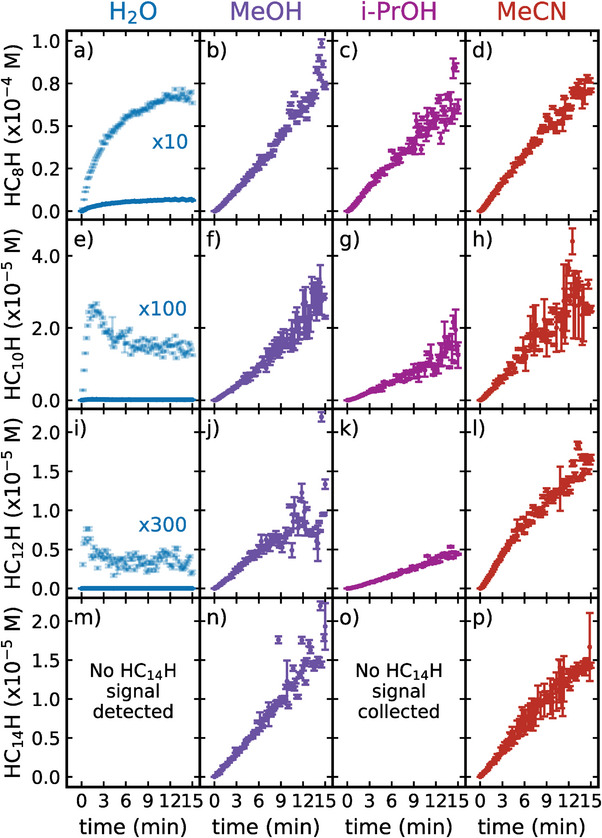
Evolution of the concentration of size‐selected H‐capped polyynes during PLAL in different solvents as a function of the ablation time. The error bars derive from the fitting errors in determining α mode and Raman solvent's area and calculation of the conversion factor. Concentration behavior of HC_8_H extracted from SA‐corrected in situ UVRR data collected at 226 nm in a) water, b) methanol, c) isopropanol, and d) acetonitrile. Concentration behavior of HC_10_H extracted from SA‐corrected in situ UVRR data collected at 251 nm in e) water, f) methanol, g) isopropanol, and h) acetonitrile. Concentration behavior of HC_12_H extracted from SA‐corrected in situ UVRR data collected at 272 nm in i) water, j) methanol, k) isopropanol, and l) acetonitrile. Concentration behavior of HC_14_H extracted from SA‐corrected in situ UVRR data collected at 264 nm in n) methanol and p) acetonitrile. No signal of HC_14_H was detected in water (panel m), while we did not collect any signal at 264 nm in isopropanol (panel o). The concentrations of HC_8_H, HC_10_H, and HC_12_H during ablation in water were multiplied by, respectively, 10 (panel a), 100 (panel e), and 300 (panel i) to help their visualization.

**Figure** **3** illustrates the evolution of the concentration of size‐selected H‐capped polyynes during ablation in various solvents, i.e., water, methanol, isopropanol, and acetonitrile. These results derive from in situ integrated UVRR signals, shown in Figure 2 and Figures S2–S4 in the Supporting Information, as corrected for the SA effect using Equation 1. The conversion into concentration was performed using Equation 2.

We use concentration dynamics from ablations in water as a negative control to highlight the advantages of organic solvents and study possible degradation pathways. Indeed, our data confirms water as the least favorable environment for the growth of polyynes by PLAL, consistent with other ex situ data.^[^ ^36^, ^37^, ^90^, ^91^, ^92^ ^]^ The concentrations reached in water for HC_8_H, HC_10_H, and HC_12_H were 1 or 2 orders of magnitude lower than in organic solvents (see Figure 3). This significant disparity underscores the importance of organic solvents as additional carbon sources for polyynes’ formation. Specifically, water's inability to provide additional carbon atoms during plasma phase dissociation inhibits the polymerization reaction of polyynes.^[^ ^36^, ^37^, ^93^ ^]^ A recent work studied cyanopolyyne's formation from an ablation in acetonitrile, using ^13^C‐enriched carbon powder and NMR analyses, showed that more than one‐fourth of the carbon in cyanopolyynes derives from solvent's dissociation.^[^ ^41^ ^]^ Our results qualitatively confirm this finding, as water's production rate underperforms organic solvents, as described in the following.

Moreover, the high thermal conductivity of water (0.6062 W m^−1^ K^−1^ at 298 K)^[^ ^94^ ^]^ compared to organic solvents such as MeOH (0.202 W m^−1^ K^−1^), i‐PrOH (0.135 W m^−1^ K^−1^), and MeCN (0.188 W m^−1^ K^−1^)^[^ ^94^ ^]^ may contribute to the lower yield of polyynes observed in water ablations. This difference could result in a less confined plasma plume during the laser ablation, potentially reducing the formation rate of polyynes. Indeed, polyynes’ synthesis requires strong out‐of‐equilibrium conditions and a high density of reactive carbon species that may be less favorable in a less dense plasma environment. Similar trends were observed with longer polyynes synthesized in propane gas than liquid hexane.^[^ ^81^ ^]^ The lower yield in a gaseous environment can be attributed to the less dense plasma plume created by the lower pressure of propane gas compared to liquid hexane, as well as the lower thermal conductivity (propane gas 0.0185 W m^−1^ K^−1^ and liquid hexane 0.1167 W m^−1^ K^−1^).^[^ ^94^ ^]^ However, the direct impact of thermal conductivity on polyynes’ synthesis dynamics remains unclear,^[^ ^50^ ^]^ and further studies are needed to validate these hypotheses.

Interestingly, the growth of HC_8_H displayed in Figure 3a in water follows a step‐like response, i.e., increasing in time and reaching a saturation concentration after ≈12 min of ablation. We rationalized this behavior by fitting with the following equation:

(3) $$c\;\left(t\right)=c_{\infty }\;\left[1-e^{-t/\tau }\right]$$

where $c_{\infty }$ (in mol/L) is the saturation concentration for t→∞ and τ (in min) is the characteristic time of the growth of this chain.

Instead, HC_10_H and HC_12_H exhibit a rapid first growth within the first 1–2 min of ablation, followed by a slower decrease toward a constant value, as illustrated in Figure 3e,i. Then, the general equation for the growth of these two chains in water can be approximated as

(4) $$c\;\left(t\right)=c_{\infty }\;\left[1-e^{-t/\tau }\right]\cdot c_{1}\left[1+e^{-\left(t-t_{0}\right)/\tau_{1}}\right]$$

where now $c_{1}$ (nondimensional), $t_{0}$ (min), and $\tau_{1}$ (min) are fitting parameters such that $c_{\infty }\cdot c_{1}$ is the new saturation concentration for t→∞. Results of the fit are reported in Figure S12 (Supporting Information).

The concentration of the different chains converges to comparable values at the end of the ablations, regardless of the organic solvent employed. However, distinct growth dynamics are observed when comparing alcohols and acetonitrile. Concerning ablations in alcohols (MeOH and i‐PrOH), the production of polyynes by PLAL follows an almost linear increase during the entire 15 min of ablation, as shown in Figure 3. We used a linear model to represent these dynamics, given by

(5) $$c\;\left(t\right)=\frac{c_{\infty }}{\tau }t$$

The slightly lower yield shown in i‐PrOH for HC_10_H and HC_12_H may be related to a bathochromic effect, i.e., the solvent‐induced shift of polyynes’ vibronic sequence toward longer wavelengths. UV‐Vis spectra in Figure S8 (Supporting Information) show the 0‐0 peaks of HC_10_H and HC_12_H in i‐PrOH at 252 nm and 276 nm, respectively, compared to 251 nm and 274 nm, respectively, in MeOH and MeCN. This minor bathochromic shift reduces the resonance enhancement and affects the absolute concentration values obtained from our conversion model.

In contrast, the growth dynamics of polyynes in MeCN deviate from linearity at longer ablation times, especially for longer chains, though these deviations are less pronounced than in water. For this reason, we modeled the growth of polyynes in MeCN with a single‐characteristic‐time model (Equation 3) to extract the relevant growth parameters.

Figure S12 (Supporting Information) presents the results of the fits applied to SA‐corrected concentration dynamics in all solvents. In summary, Equation 3 has been used to model the behavior of HC_8_H in water and HC_n_H (n = 8‐14) in MeCN, Equation 4 can catch the dynamics of HC_10_H and HC_12_H in water, while Equation 5 represents the evolution of HC_n_H (n = 8‐14) in MeOH and i‐PrOH.

**Figure 4:**
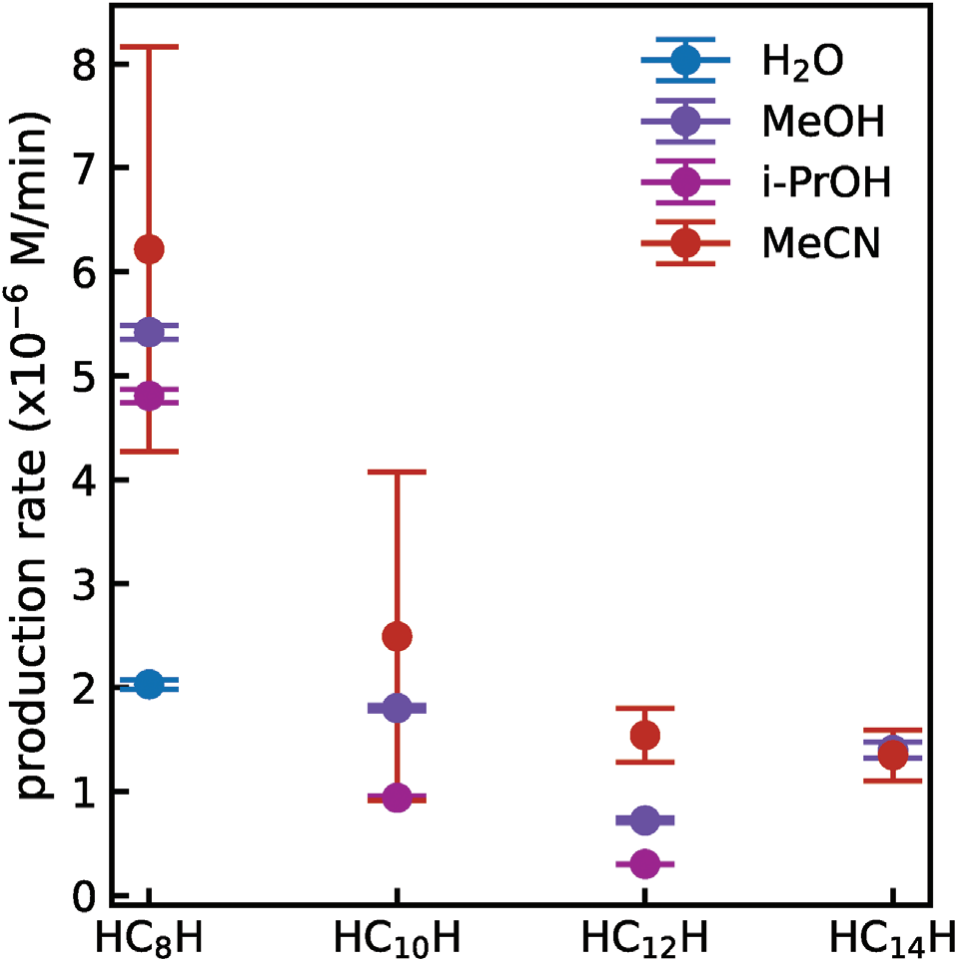
Production rates of H‐capped polyynes (HC_n_H, n = 8‐14) extracted from fitting procedures of the evolution of the corresponding concentration curves (see Figure S12, Supporting Information). Numerical values are reported in Table S4, (Supporting Information). The error bars originate from the fit error. The production rates of HC_10_H and HC_12_H in water cannot be retrieved from the fitting model used.

The term $c_{\infty }$ in Equation 5 can be interpreted as the production rate of H‐capped polyynes, expressed in M min^‐1^ (where M is molarity, i.e., mol L^−1^), which calculated values are displayed in **Figure** **4** (numerical values are reported in Table S4, Supporting Information). This parameter provides an effective metric for evaluating the growth dynamics across various solvents. In the case of acetonitrile and water, when the growth is modeled using Equation 3, we can approximate the single‐characteristic‐time model to its first‐order Taylor expansion term, yielding the expression $c\left(t\right)\;\approx \;\frac{c_{\infty }}{\tau }t$ ‒, i.e., Equation 5. However, this approximation does not hold for the growth dynamics of HC_10_H and HC_12_H in water. The production rate estimated for HC_14_H in MeOH is larger than for HC_12_H. This effect may be related to the more scattered concentration values for HC_14_H, as reported in Figure 3n and Figure S12m (Supporting Information). The production rates in organic solvents are comparable, while those in water drop, confirming the low productivity when ablating in this environment. Excluding from our considerations the production rates of i‐PrOH, for which the bathochromic effect introduces some distortions, and the scattered values for HC_14_H, we notice a slight increase in the production rates passing from MeOH to MeCN.

The findings presented in Figures 3 and 4 enable us to review the growth dynamics of polyynes produced by PLAL across different solvents. The observed saturation at longer ablation times suggests that a peculiar formation‐degradation equilibrium exists between the formation of polyynes by the PLAL and their degradation mechanisms. Degradation reactions are necessary to achieve this equilibrium since no evidence supports different production efficiencies at longer ablation times, as discussed in our previous works.^[^ ^36^, ^73^ ^]^ The most probable degradation pathways are oxidation and crosslinking reactions. We can exclude photo‐induced degradation due to the lower energy of PLAL photons compared to polyynes' electronic transitions, while thermal‐induced degradation is unlikely given the stable solution temperature of 300 K observed during similar experiments.^[^ ^36^, ^37^, ^73^ ^]^

Instead, water's and alcohol's dissociation under laser irradiation can produce molecular, atomic, or radical oxygen, detrimental to polyynes’ stability by inducing oxidation reactions.^[^ ^36^, ^37^, ^80^, ^92^, ^93^ ^]^ Conversely, MeCN cannot dissociate in such species. Furthermore, MeCN possesses lower dissolved oxygen levels than the other solvents ‒ one and two orders of magnitude less than water and alcohols, respectively.^[^ ^95^, ^96^ ^]^ However, the fast diffusion of molecular oxygen in water and organic solvents^[^ ^97^ ^]^ prevents its accumulation, thus making it improbable to be responsible for long‐term degradation reactions. Atomic and radical oxygen are highly reactive and react during the plasma phase (at most 100 ns after the ablation event) before polyynes can be detected by the in situ probe. These points indicate that degradation via oxidation is less significant, which is supported by the observed linear growth in alcohols where radical oxygen can be produced and molecular oxygen readily dissolves.

In contrast, the crosslinking probability increases with higher concentrations of polyynes and byproducts. In water, a highly polar solvent (relative polarity 1, where the polarity is defined as in Refs. [83, 84]), the limited solubility of polyynes accelerates their aggregation, hastening degradation through crosslinking. This results in reaching the saturation concentration more rapidly, as observed for HC_8_H (Figure 3a), than in organic solvents. For HC_10_H (Figure 3e) and HC_12_H (Figure 3i), this process leads to a saturation regime within less than 3 min, with a saturation concentration lower than the peak concentration.

Instead, polyynes exhibit higher solubility in organic solvents, whose relative polarity increases from MeCN (0.46) to i‐PrOH (0.564) to MeOH (0.762).^[^ ^83^, ^84^ ^]^ Despite MeCN's lower polarity compared to alcohols, it reaches the formation‐degradation equilibrium faster. This accelerated equilibrium is due to the larger production rate of byproducts in MeCN compared to alcohols (Figure 4). MeCN's tendency to carbonization upon dissociation^[^ ^37^, ^82^ ^]^ and its higher C/H ratio (0.67) ‒, i.e., the balance between carbon and hydrogen atoms in solvent molecules ‒ relative to alcohols (0.33 for MeOH and 0.5 for i‐PrOH) contribute to this greater production rate. The solvent's C/H ratio plays a pivotal role in determining polyynes’ nucleation.^[^ ^37^, ^81^, ^93^, ^98^ ^]^ These characteristics promote an extensive formation of carbonaceous compounds, including polyynes and byproducts, thereby enhancing the production rate while increasing crosslinking probability. The higher production of byproducts is evident by the dark brown color of MeCN mixtures, in contrast to yellow and orange solutions for MeOH and i‐PrOH, respectively.^[^ ^37^ ^]^ This heightened degradation in MeCN contrasts with our previous observation that identified MeCN as the most stable environment among those tested ‒ water, MeOH, i‐PrOH, ethanol, and MeCN ‒ for polyynes’ stability.^[^ ^37^ ^]^ However, those results are based on ablations in MeCN using a 532 nm laser, which produces fewer byproducts than ablations at 1064 nm, indicated by a lighter MeCN mixture color (i.e., dark brown for 1064 nm and dark orange for 532 nm). Therefore, differences in production rates of both byproducts and polyynes can justify this discrepancy, emphasizing the importance of considering the wavelength effect on polyynes’ growth dynamics in future studies.

Alcohols, as mentioned above, yield cleaner solutions with lower levels of byproducts. This reduces the probability of crosslinking reactions, resulting in linear growth (Figure 3). Despite their higher polarity than MeCN, crosslinking reactions show an enhanced correlation to the concentration of carbonaceous products within the mixture than increased aggregation due to high polarity. Like other solvents, we anticipate that alcohols will eventually reach a formation‐degradation equilibrium, albeit at longer ablation times. The higher hydrogen content or lower C/H ratio in alcohols can stimulate termination reactions that stabilize polyynes while increasing the formation of byproducts such as hydrocarbons. Therefore, while the substantial C/H ratio of alcohols is pivotal in determining their high production rate (Figure 4), it is not the sole solvent property influencing this parameter.

**Figure 5:**
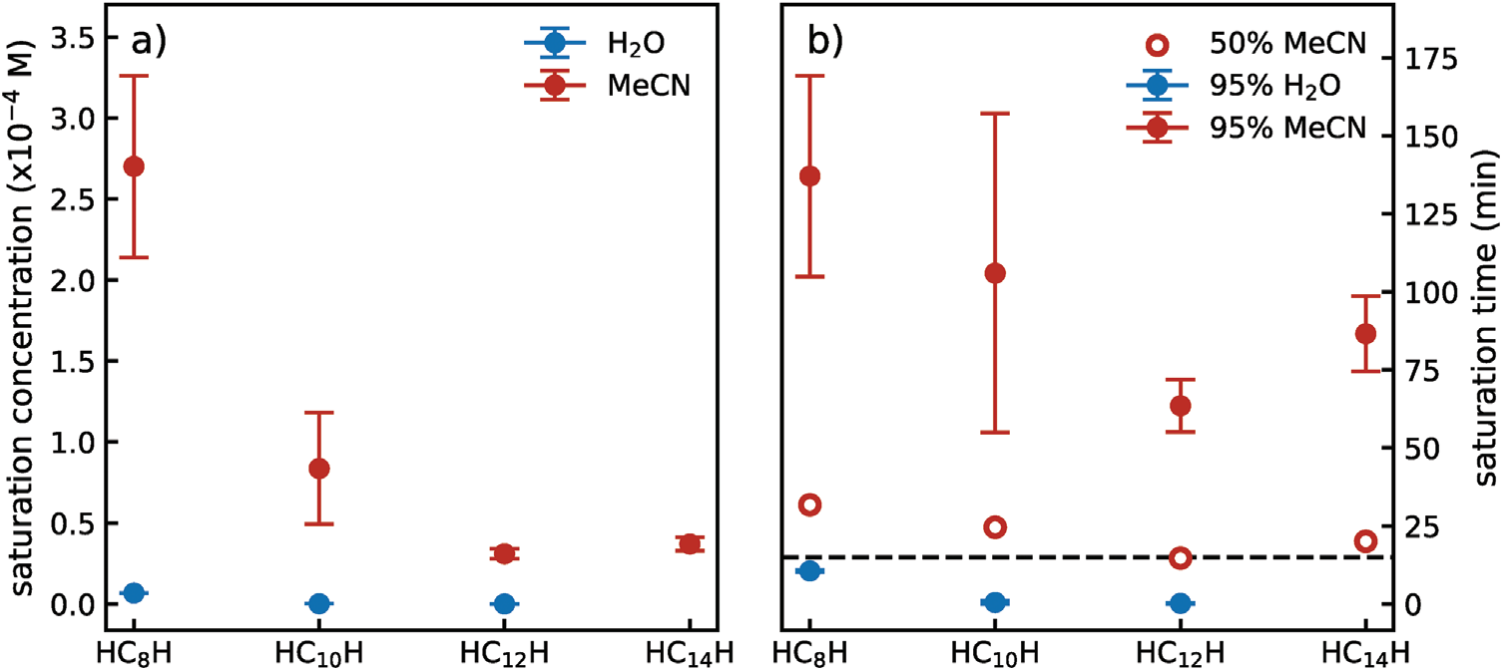
Saturation concentration and time of H‐capped polyynes from PLAL in water and acetonitrile. a) Saturation concentration (in M) and b) saturation time to reach 50% (open circles) and 95% (filled circles) of the saturation concentration (in min) extracted from the fitting procedures of the evolution of the concentration curves in water and acetonitrile (see Figure S12, Supporting Information). The horizontal dashed line in panel b highlights the 15 min value. Numerical values are reported in Table S5 (Supporting Information). The fit errors are shown as well.

For the solvents featuring formation‐degradation equilibrium within 15 min of ablation (such as water) or showing a tendency toward it (like MeCN), we present in **Figure** **5**a the saturation concentrations ($c_{\infty }$ from Equation 3 or $c_{\infty }c_{1}$ from Equation 4) achieved in our ablation conditions. Consequently, we display in Figure 5b two saturation times, defined as the ablation time needed to arrive at 50% (open circles) and 95% (filled circles) of the saturation concentration. These parameters serve as a practical measure to discuss polyynes’ growth dynamics. The almost linear growth of shorter chains (HC_8_H and HC_10_H) in MeCN introduces larger errors in determining the saturation concentration and time. The saturation concentration and saturation time for HC_14_H appear higher than HC_12_H's, but these values might be inaccurately predicted due to the noisier in situ UVRR spectra recorded at 264 nm, as shown by the larger error bars in Figure 3p.

These parameters decrease with increasing chain length, reflecting the rapid reduction in the probability of longer polyynes’ synthesis and their weaker stability as the chain length extends. They highlight even more the solvent role in providing additional carbon atoms. Indeed, the saturation concentrations achieved in ablations in acetonitrile overcome those in water by 2 or 3 orders of magnitude. In parallel, the saturation times to achieve 95% saturation concentration in water are relatively short, ≈10 min for HC_8_H and 26 and 8 s for HC_10_H and HC_12_H, respectively. These values mirror the insolubility and consequent instability of polyynes in water (see Figure 5b and Table S4, Supporting Information). Conversely, MeCN features much longer saturation times that easily overcome 1 h of ablation (see Figure 5b and Table S4, Supporting Information). Instead, the ablation time to reach 50% of saturation concentration tells us that within the duration of our in situ UVRR experiments, we are close to these concentration values for all polyynes.

Concerning alcohols, i.e., MeOH and i‐PrOH, even though we cannot perform these kinds of estimations, we can predict their behavior at longer ablation times. Indeed, their lower tendency to carbonization suggests higher saturation concentrations and times in alcohols than those observed in MeCN.

Interestingly, we can predict the saturation concentration of longer H‐capped polyynes, which cannot be probed due to the wavelength limitations of the beamline, starting from the data from ablations in MeCN reported in Figure 5a. Indeed, the size‐dependent behavior of the saturation concentration in Figure 5a can be accurately fit with an exponential function. Table S6 (Supporting Information) reports the expected saturation concentrations for wires from HC_16_H up to HC_30_H, i.e., the longest H‐capped polyyne ever detected produced by PLAL,^[^ ^45^ ^]^ resulting in ≈10^−9^ mol L^−1^. Despite the low concentration, the enhancement coming from resonance Raman and the increasing Raman activity of polyynes with the chain length (refer to the Supporting Information of Ref. [73]) could, in principle, allow us to detect this chain by collecting in situ UVRR spectra at 406 nm, i.e., the wavelength of its 0‐0 vibronic transition^[^ ^45^ ^]^ However, this wavelength and the others needed to perform in situ UVRR experiments for chains longer than HC_14_H are far beyond the available range of the IUVS beamline. Further experiments are required to assess the growth of longer chains as the new generation of tunable lasers may represent a valid alternative to provide sufficient tunability in this UV region (270 – 400 nm) approaching the visible range.

These results indicate the unnecessariness of longer ablations due to an upper limitation in polyynes’ concentration in a limited volume mixture (i.e., 2 mL in this work). This limit arises from degradation mechanisms, particularly crosslinking, which reduces the ablation efficiency over time. Therefore, alternative approaches should be explored to achieve polyynes’ concentrations required for applications or characterizations with less sensitive techniques. These include performing ablations in larger liquid volumes or using continuous flow systems, thus applying post‐ablation treatments, such as concentrating the solution to a smaller volume. Our findings also showed that solvents with a high C/H ratio and a tendency to carbonization upon dissociation tend to reach the formation‐degradation equilibrium faster due to an enhanced production rate. This can result in poorer polyynes’ storage stability and may necessitate filtration and purification steps after the ablation. Alternatively, transferring polyynes to stabilizing environments, such as encapsulating them in polymeric films or nanotubes, can stabilize them.

## 3 Conclusions

The in situ UV resonance Raman approach presented in this study represents a unique and direct method to monitor carbon atomic wires’ synthesis in liquid. It allows for real‐time, direct monitoring of chains’ growth during PLAL experiments without perturbing the synthesis process or the ablation environment. Exploiting the fine tunability of synchrotron‐based UV radiation, we achieved unprecedented size selectivity, enabling us to track the single‐chain species growth dynamics. Moreover, the sensitivity of the in situ UVRR probe, enhanced by matching the resonance condition, allowed us to detect and study the whole dynamics of wires’ fabrication, even at the very early stages where their concentrations were down to 10^−9^‐10^−10^ mol L^−1^.

We delved into the effect of diverse solvents in our in situ PLAL experiments, including water, methanol, isopropanol, and acetonitrile. This comprehensive and systematic approach allowed us to gain unprecedented insights into carbon atomic wires’ growth dynamics through PLAL. By developing an empirical model to correct UVRR data from self‐absorption and converting them into concentration values, we gained a deeper understanding of these dynamics.

Our modeling of the growth dynamics of carbon atomic wires during PLAL revealed size‐dependent production rates, which range from ≈10^−6^ to ≈10^−7^ M min^−1^, meaning a size‐selected H‐capped polyyne average production of ≈10^11^–10^12^ wires per pulse with a 10 Hz laser (i.e., 600 shots per minute) in 2 mL of solution. These production rates are influenced by the solvent's properties, with carbon‐rich solvents that tend to carbonize under dissociation resulting in higher production rates.

We unveiled that the growth did not follow a linear behavior. Instead, the concentration of carbon atomic wires tends to reach an equilibrium state between formation and degradation, primarily due to crosslinking reactions. This equilibrium results in a saturation concentration that decreases and is reached more quickly as chain length increases, indicating a reduced synthesis yield and stability for longer chains.

Organic solvents yielded saturation concentrations that were orders of magnitude higher and took significantly longer times to reach equilibrium compared to water. We think that future studies should consider using ^13^C‐enriched solvents as a quantitative way to evaluate the solvent's contribution in providing additional carbon atoms for polyynes polymerization. Our findings demonstrated that performing single, longer (> 1 h) ablations in small liquid volumes is not effective for obtaining high concentrations of carbon atomic wires through PLAL. Instead, alternative approaches must be explored to overcome these limitations.

Our findings demonstrate the importance of in situ characterization techniques in providing valuable insights into the growth dynamics of carbon atomic wires and, more in general, in enhancing our ability to study and manipulate materials at the atomic and molecular levels. In particular, the exceptional versatility and scalability of PLAL provide the foundation for tailoring sp‐carbon chains’ structure and achieving scale‐up production of carbon atomic wires by PLAL. This will unlock the possibility of applying sp‐carbon chains in a wide range of applications beyond the existing prototypal devices^[^ ^99^, ^100^, ^101^, ^102^, ^103^ ^]^ that showcase the outstanding potential of this material.

## 4 Experimental Section

### Pulsed Laser Ablation in Liquid

Pulsed laser ablation in liquid (PLAL) was performed using a ns‐pulsed Nd:YAG laser (Quantel Q‐Smart 850, repetition rate 10 Hz), as shown in Figure 1. The fundamental harmonic of the Nd:YAG laser at 1064 nm, with a pulse duration of 5 ns, was employed for all ablations. The laser energy was fixed to 50 mJ per pulse through a beam attenuator module equipped with the laser head. The laser beam was focused with a lens with a 200 mm focal length (see Figure 1b) onto a graphite target (Testbourne Ltd., purity 99.99%). Ablations were performed for 15 min in 2 mL of different solvents: deionized water (H_2_O) Milli‐Q (conductivity 0.055 µS), methanol (MeOH, Sigma‐Aldrich, purity ≥99.9%), isopropanol (i‐PrOH, Sigma‐Aldrich, purity ≥99.9%), and acetonitrile (MeCN, Sigma‐Aldrich, purity ≥99.9%).

The target and the solvent were placed into a PTFE ablation cell, prepared *ad hoc* for these experiments and schematically reported in Figure 1b. The cell features a cylindrical hole, opened at the top, allowing the PLAL laser to enter, and two quartz windows (Hellma, QS UV‐Vis range 200–2500 nm), through which the monochromatized synchrotron radiation was focused to collect UV resonance Raman spectra during ablation experiments. The liquid volume reached a height of ≈18.7 mm from the target surface inside the ablation cell, enough to completely cover the quartz windows. The target‐to‐lens distance was set to ≈177.9 ± 0.6 mm by fixing the ablation cell on a steel pillar.

### UV Resonance Raman Spectroscopy

In situ UV resonance Raman (UVRR) spectra were acquired using the synchrotron‐based UVRR setup accessible at the BL10.2‐IUVS beamline of Elettra Sincrotrone (Trieste, Italy) and described in detail here.^[^ ^104^ ^]^ Various excitation wavelengths in the deep UV range were employed, as detailed in Table S1 of the Supporting Information (SI), by precisely adjusting the undulator gap aperture to tune the energy of the emitted synchrotron radiation (SR). Subsequently, the SR light was monochromatized via a 750 cm focal length spectrograph equipped with a holographic grating featuring 3600 grooves/mm. It is worth noting that UV excitation beyond 272 nm or below 200 nm is not possible due to the limitations imposed by the minimum undulator gap aperture and by the optical elements of the monochromator. The incident radiation power on the samples varied, ranging from a few to tens of µW (see Table S1, Supporting Information). Spectrometer calibration was executed using the spectrum of cyclohexane (spectroscopic grade, Sigma Aldrich). The ultimate spectral resolution was influenced by several factors, including the SR monochromator's resolving power (determined by factors such as focal length, grating, and slit aperture), the excitation wavelength, and the spectral range as constrained by the pixel spacing of the CCD. For each excitation wavelength, the final resolution of the recorded Raman spectra could be reliably estimated using the following general equation $\frac{spectral\;range\;[cm^{-1}]}{1340}$. As an illustration, resolutions of 2.6, 1.9, and 1.6 cm^−1^ per pixel were achieved at 216, 251, and 272 nm excitation wavelengths, respectively. The spectra were all collected at room temperature.

## Conflict of Interest

The authors declare no conflict of interest.

## Author Contributions

P.M., S.P., A.L.B., V.R., and C.S.C. conceived the experiment. P.M., S.P., B.R., A.G., and C.S.C. carried out in situ UV resonance measurements. A.G. designed and realized the ablation cell. P.M., S.P., S.M., B.R., and V.R. carried out ex situ UV resonance Raman measurements and all the other ex situ measurements. P.M., S.P., and S.M. performed data analysis. P.M. wrote the first version of the paper. All authors discussed the results and contributed to writing subsequent manuscript drafts.

## Supporting information

Supporting Information

## Data Availability

The data that support the findings of this study are available from the corresponding author upon reasonable request. They have been deposited in Zenodo with this identifier https://doi.org/10.5281/zenodo.10843187.
