# Supplementary material for: Exploring the Growth Dynamics of Size‐Selected Carbon Atomic Wires with In Situ UV Resonance Raman Spectroscopy
Source: Small. 2024 Jul 28;20(49):2403054. doi: 10.1002/smll.202403054 (PMC11618744; doi:10.1002/smll.202403054)
Supplement: Supplementary file 1 — Supporting Information [file SMLL-20-2403054-s001.docx]

**Supporting Information**

Exploring the Growth Dynamics of Size-selected Carbon Atomic Wires with *in situ* UV Resonance Raman Spectroscopy

Pietro Marabotti^a,b*^, Sonia Peggiani^a^, Simone Melesi^a^, Barbara Rossi^c^, Alessandro Gessini^c^, Andrea Li Bassi^a^, Valeria Russo^a^, Carlo Spartaco Casari^a*^

*^a^ Department of Energy, Micro and Nanostructured Materials Laboratory - NanoLab, Energy, Politecnico di Milano, Via Ponzio 34/3, Milano 20133, Italy*

*^b^ Institut für Physik, Humboldt Universität zu Berlin, 12489 Berlin, Germany*

*^c^ Elettra Sincrotrone Trieste, S.S. 114 km 163.5, Basovizza, 34149 Trieste, Italy.*

*Corresponding authors: [pietro.marabotti@polimi.it](mailto:pietro.marabotti@polimi.it), [carlo.casari@polimi.it](mailto:carlo.casari@polimi.it)


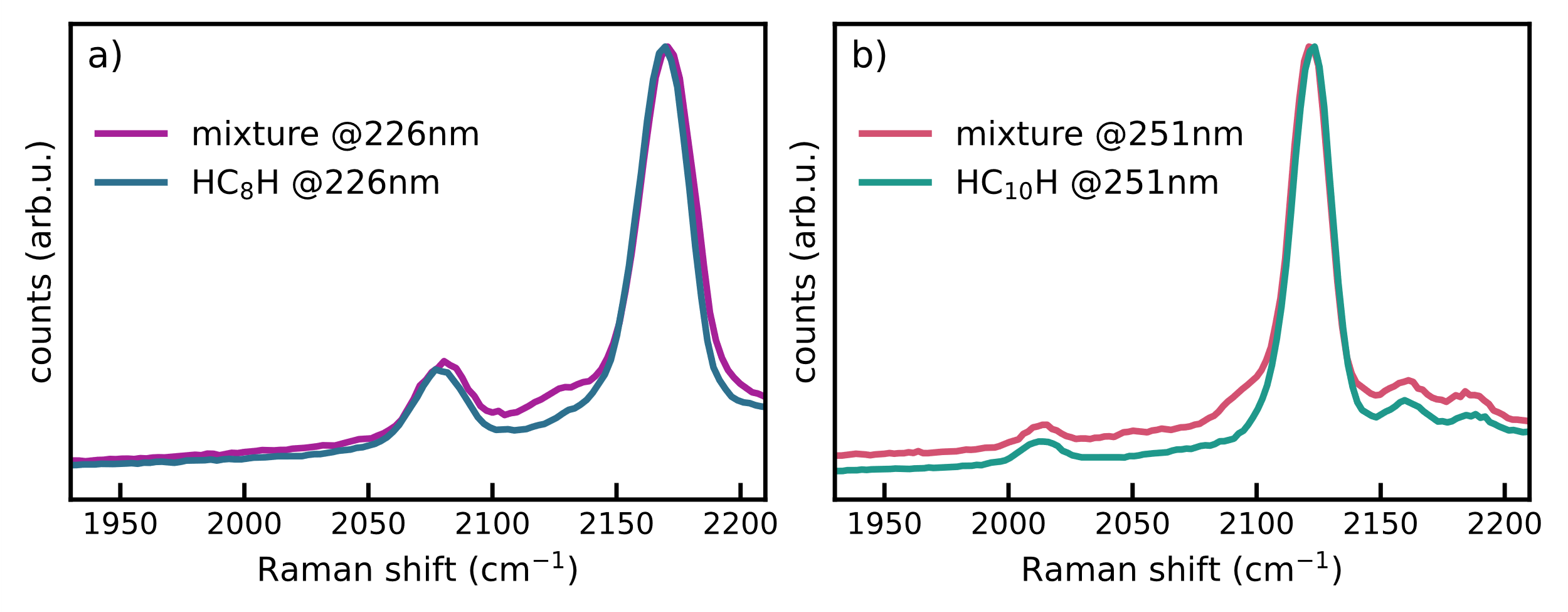


**Figure S1 Comparison between mixture and size-selected UVRR spectra of polyynes.** a) UVRR spectra of a mixture of polyynes (purple curve) and a size-selected H-capped polyyne (HC_8_H, blueish curve) collected at 226 nm as excitation wavelength. b) UVRR spectra of a mixture of polyynes (pinkish curve) and a size-selected H-capped polyyne (HC_10_H, blueish curve) collected at 251 nm as excitation wavelength.

| **Polyyne** | **Excitation wavelength [nm]** | **Power on the sample [μW]** | **Aperture slits [μm]** | **Acquisition time [s]** | **CN stretching mode area (pristine solution)** |
| --- | --- | --- | --- | --- | --- |
| HC_8_H | 226 | 16.9 | 50 | 10 | 27178 ± 67 |
| HC_10_H | 251 | 18.6 | 50 | 10 | 14094 ± 28 |
| HC_12_H | 272 | 8.7 | 30 | 10 | 34832 ± 51 |
| HC_14_H | 264 | 14.7 | 30 | 10 | 90861 ± 111 |

**Table S1** *In situ* UVRR parameters employed to monitor polyynes’ growth during pulsed laser ablation in liquid (PLAL) experiments for each polyyne chain. CN stretching mode’s UVRR integrated signal extracted before ablations in acetonitrile is reported.


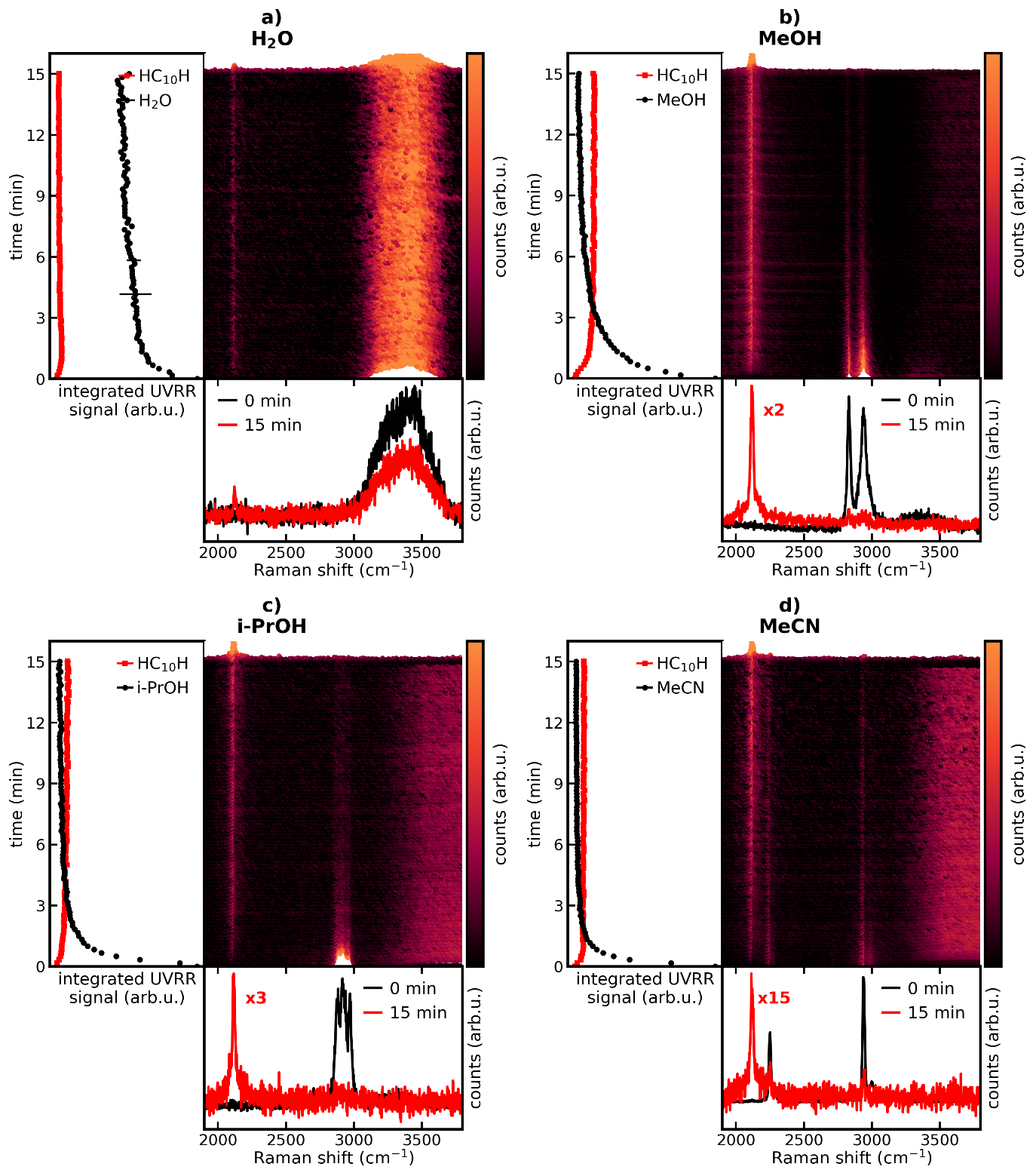


**Figure S2 In situ UVRR spectra at 251 nm during ablations in different solvents.** UVRR spectra collected at 251 nm Raman excitation in a) water, b) methanol (MeOH), c) isopropanol (i-ProH), and d) acetonitrile (MeCN) during 15 minutes of ablation (1064 nm ablation laser, 15 minutes of ablation time, 50 mJ per pulse), in the colormap of each panel. The integrated UVRR Raman signals of HC_10_H’s α mode (red squares) and the relevant solvent Raman band (black circles, see main text) are displayed in the left-hand box of each panel. Fit errors (see Section S1) are shown with error bars. The first (0 min) and last (15 min) spectra are reported in the bottom box of each panel. The 15 min spectra are multiplied by a variable factor to improve the visualization.


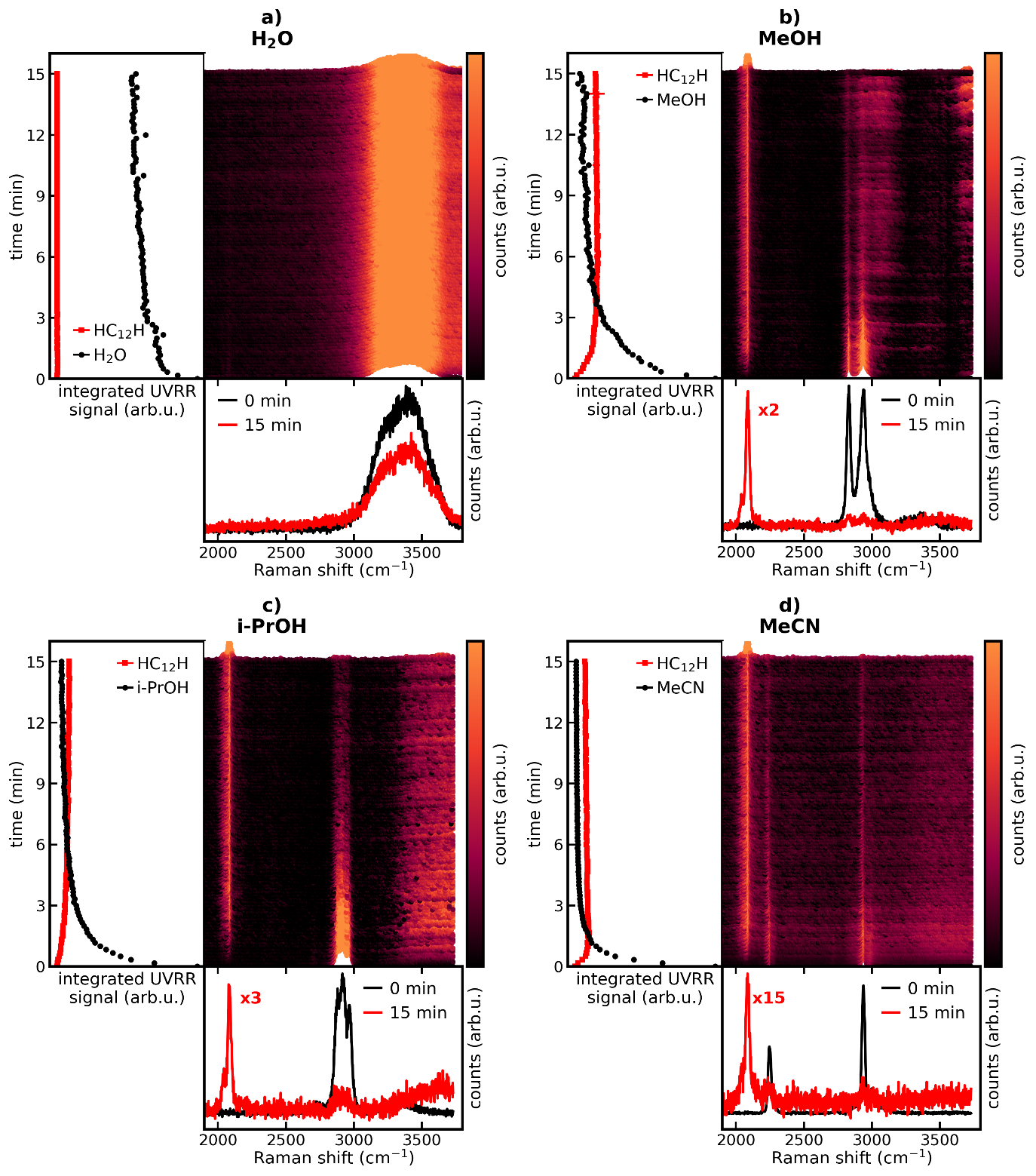


**Figure S3 In situ UVRR spectra at 272 nm during ablations in different solvents.** UVRR spectra collected at 272 nm Raman excitation in a) water, b) methanol (MeOH), c) isopropanol (i-ProH), and d) acetonitrile (MeCN) during 15 minutes of ablation (1064 nm ablation laser, 15 minutes of ablation time, 50 mJ per pulse), in the colormap of each panel. The integrated UVRR Raman signals of HC_12_H’s α mode (red squares) and the relevant solvent Raman band (black circles, see main text) are displayed in the left-hand box of each panel. Fit errors (see Section S1) are shown with error bars. The first (0 min) and last (15 min) spectra are reported in the bottom box of each panel. The 15 min spectra are multiplied by a variable factor to improve the visualization.


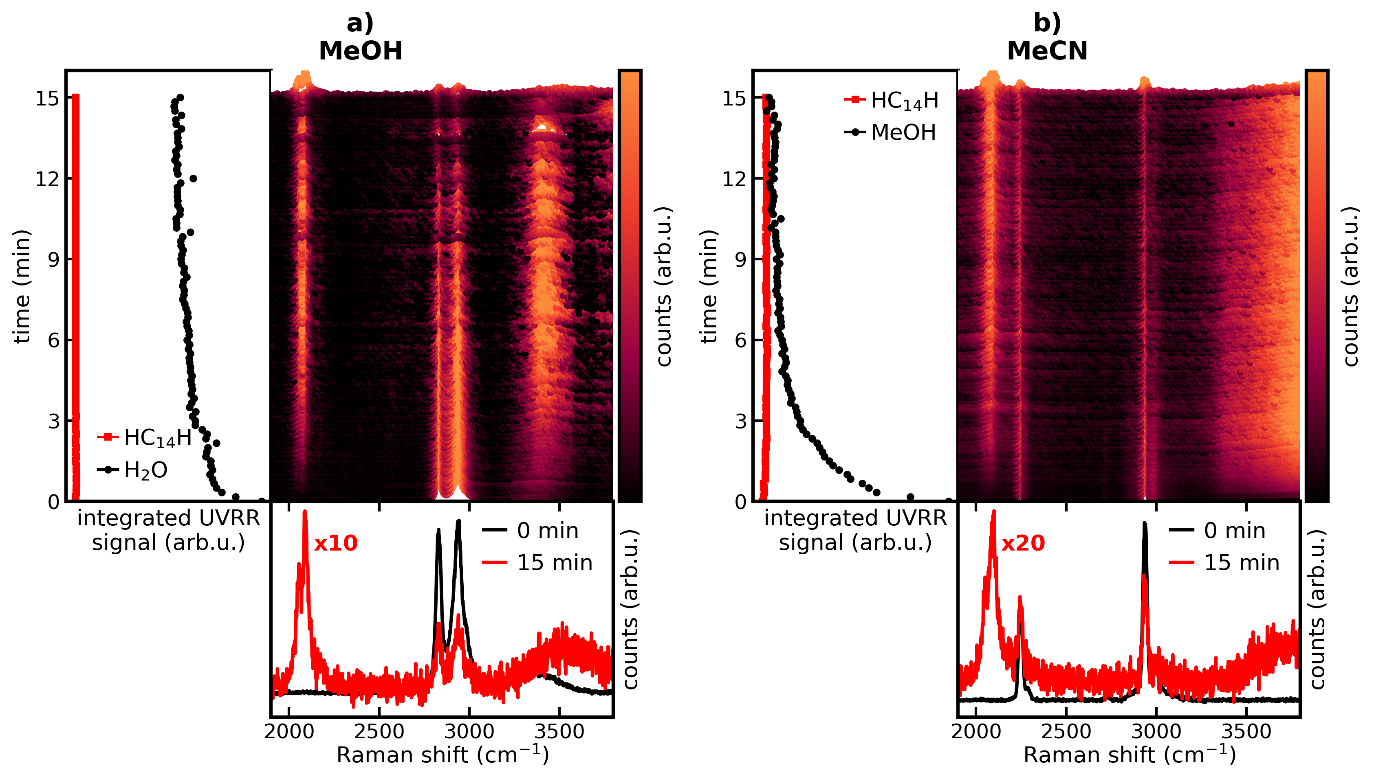


**Figure S4 In situ UVRR spectra at 264 nm during ablations in different solvents.** UVRR spectra collected at 264 nm Raman excitation in a) methanol (MeOH) and b) acetonitrile (MeCN) during 15 minutes of ablation (1064 nm ablation laser, 15 minutes of ablation time, 50 mJ per pulse), in the colormap of each panel. The integrated UVRR Raman signals of HC_14_H’s α mode (red squares) and the relevant solvent Raman band (black circles, see main text) are displayed in the left-hand box of each panel. Fit errors (see Section S1) are shown with error bars. The first (0 min) and last (15 min) spectra are reported in the bottom box of each panel. The 15 min spectra are multiplied by a variable factor to improve the visualization.


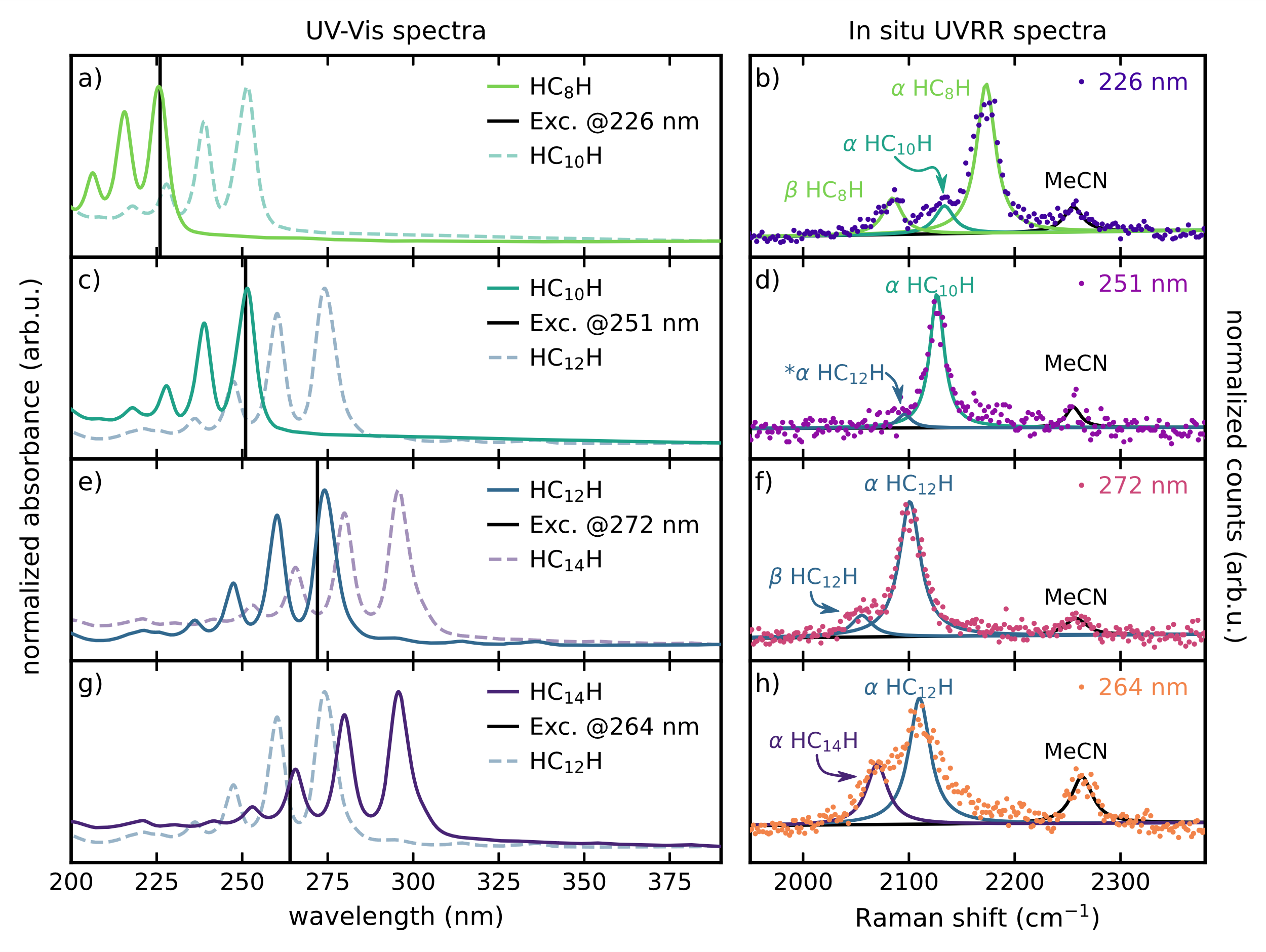


**Figure S5 Resonace conditions from UV-Vis spectra and assignment of spectra features in UVRR spectra of H-capped polyynes.** a) UV-Vis spectra of HC_8_H (solid line) and HC_10_H (semi-transparent dashed line). The black solid line at 226 nm, crossing the 0-0 and 0-2 vibronic peaks of HC_8_H and HC_10_H, respectively, indicates the Raman excitation wavelength employed to record *in situ* UVRR spectrum in panel b. b) *In situ* UVRR spectrum collected at Raman excitation 226 nm during PLAL in acetonitrile (dark purple dots). The labels mark the Raman peaks present in the spectrum. The fitted Lorentzian Raman features are reported as solid colored lines. c) UV-Vis spectra of HC_10_H (solid line) and HC_12_H (semi-transparent line). The black solid line at 251 nm, crossing the 0-0 and approximately the 0-2 vibronic peaks of HC_10_H and HC_12_H, respectively, indicates the Raman excitation wavelength employed to record *in situ* UVRR spectrum in panel d. d) *In situ* UVRR spectrum collected at Raman excitation 251 nm during PLAL in acetonitrile (purple dots). The labels mark the Raman peaks present in the spectrum. The α mode of HC_12_H is barely visible (marked with an “*”). The fitted Lorentzian Raman features are reported as solid colored lines. e) UV-Vis spectra of HC_12_H (solid line) and HC_14_H (semi-transparent dashed line). The black solid line at 272 nm, crossing the 0-0 and in between the 0-1 and 0-2 vibronic peaks of HC_12_H and HC_14_H, respectively, indicates the Raman excitation wavelength employed to record *in situ* UVRR spectrum in panel f. f) *In situ* UVRR spectrum collected at Raman excitation 272 nm during PLAL in acetonitrile (dark pink dots). The labels mark the Raman peaks present in the spectrum. The fitted Lorentzian Raman features are reported as solid colored lines. g) UV-Vis spectra of HC_14_H (solid line) and HC_12_H (semi-transparent dashed line). The black solid line at 264 nm, crossing the 0-2 and approximately the 0-1 vibronic peaks of HC_14_H and HC_12_H, respectively, indicates the Raman excitation wavelength employed to record *in situ* UVRR spectrum in panel h. h) *In situ* UVRR spectrum collected at Raman excitation 264 nm during PLAL in acetonitrile (orange dots). The labels mark the Raman peaks present in the spectrum. The fitted Lorentzian Raman features are reported as solid colored lines.

| Raman excitation [nm] | Solvent | Detected Raman shift [cm^-1^] | | |
| --- | --- | --- | --- | --- |
|  |  | **β HC_8_H**  **(weak)** | **α HC_10_H**  **(shoulder)** | **α HC_8_H**  **(strong)** |
| 226 | **Water** | 2089 | ‒ | 2179 |
|  | **MeOH** | 2083 | 2130 | 2175 |
|  | **i-PrOH** | 2084 | 2132 | 2175 |
|  | **MeCN** | 2086 | 2134 | 2176 |
|  |  | **β HC_10_H** | **α HC_12_H** | **α HC_10_H** |
| 251 | **Water** | 2018 | ‒ | 2130 |
|  | **MeOH** | 2019 | 2101 | 2127 |
|  | **i-PrOH** | 2018 | 2100 | 2126 |
|  | **MeCN** | 2021 | 2099 | 2128 |
|  |  | **β HC_12_H** | **α HC_14_H** | **α HC_12_H** |
| 272 | **Water** | ‒ | ‒ | 2106 |
|  | **MeOH** | 2052 | ‒ | 2101 |
|  | **i-PrOH** | 2053 | ‒ | 2099 |
|  | **MeCN** | 2054 | ‒ | 2100 |
|  |  | **β HC_12_H** | **α HC_14_H** | **α HC_12_H** |
| 264 | **MeOH** | ‒ | 2064 | 2102 |
|  | **MeCN** | ‒ | 2066 | 2106 |

**Table S2** Average Raman shifts of the Raman modes detected in the polyyne frequency range (1800‒2200 cm^-1^) in *in situ* UVRR data of Figure 2 (see main text), Figure S2, Figure S3, and Figure S4. The “‒” symbol indicates that a mode is not observed in the corresponding UVRR spectra. The frequencies are rescaled by a common factor for each Raman excitation wavelength, *i.e.*, 1.00482 for 226 nm, 1.00397 for 251 nm, 1.00637 for 272 nm, and 1.00525 for 264 nm. This factor makes the CN stretching mode of MeCN match its tabulated frequency, *i.e.*, 2258 cm^-1^.

# **Fitting procedure**

We employed a custom fitting procedure to model *in situ* UVRR spectra and evaluate the evolution of the different Raman bands during the ablation.

We first identify the best fitting function for each pristine solvent (see Figure S6). For the OH stretching band of water, we employed two Gaussian curves, while we used only one Gaussian curve for the OH band in alcohols (methanol and isopropanol). We selected a Lorentzian function for each peak in the CH stretching frequency region of organic solvents (methanol, isopropanol, and acetonitrile). We chose the same function for the CN stretching mode of acetonitrile. The number of curves used to fit the solvent’s Raman features has been selected to estimate their area as well as possible without completely modeling the corresponding Raman bands. To fit the solvent’s Raman peaks during *in situ* UVRR experiments, we fixed the full width at half maximum (FWHM) of each solvent’s Raman peak to the results obtained for the pristine solution. This prevents overfitting caused by numerous peaks in each spectrum.


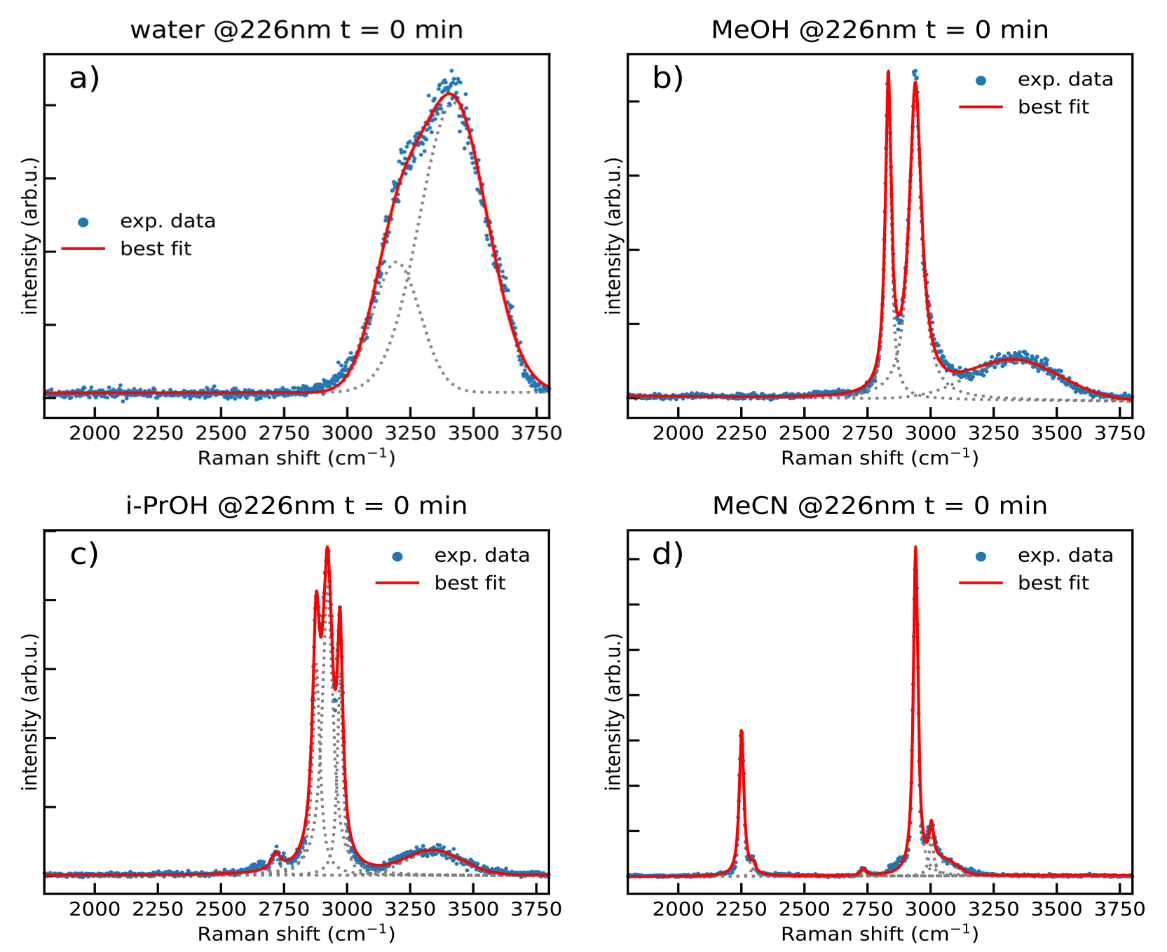


**Figure S6 UVRR spectra at 226 nm of pristine solvents.** UVRR spectra collected at 226 nm of pristine a) water, b) methanol, c) isopropanol, and d) acetonitrile solutions (blue dots). The red line represents the best fit function of each spectrum. Gray dotted lines show each component of the best fit.

In each series of *in situ* UVRR spectra, *i.e.*, excited at the same wavelength, we used a Lorentzian curve for all the polyynes’ Raman modes, namely the α and β modes of each HC_n_H polyyne excited at its 0‒0 vibronic transition (HC_8_H in Figure S5) and the α mode of the HC_n+2_H polyyne excited at its 0‒2 vibronic transition (HC_10_H in Figure S5). In particular, for each excitation wavelength, we set the FWHM of polyynes’ Raman peaks to that of the CH stretching vibration of acetonitrile at approximately 2940 cm^-1^ and did not vary them during the fitting of *in situ* UVRR data. This allowed us to use the area as the parameter of merit to monitor the evolution of polyynes’ α mode during the ablation. Figure S7 shows the results of the fitting process after 5 min of ablations in the case of *in situ* UVRR spectra collected at 226 nm, *i.e.*, in resonance with the 0-0 vibronic of HC_8_H.


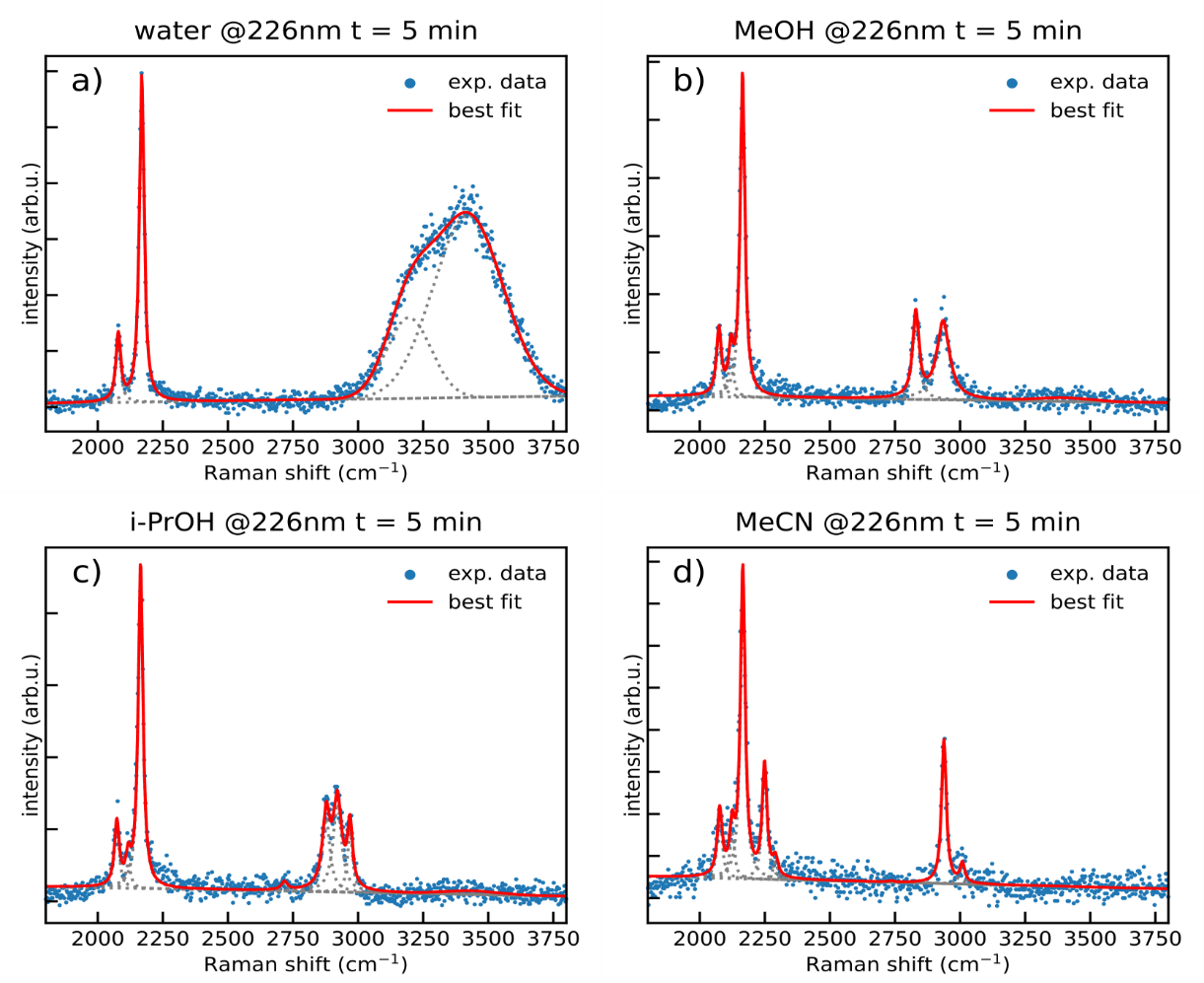


**Figure S7 In situ UVRR spectra at 226 nm after 5 min of ablations in different solvents.** *In situ* UVRR spectra collected at 226 nm after 5 min of ablation in a) water, b) methanol, c) isopropanol, and d) acetonitrile (blue dots). The red line represents the best fit function of each spectrum. Gray dotted lines show each component of the best fit.

# **Correction of self-absorption in resonance Raman spectra**


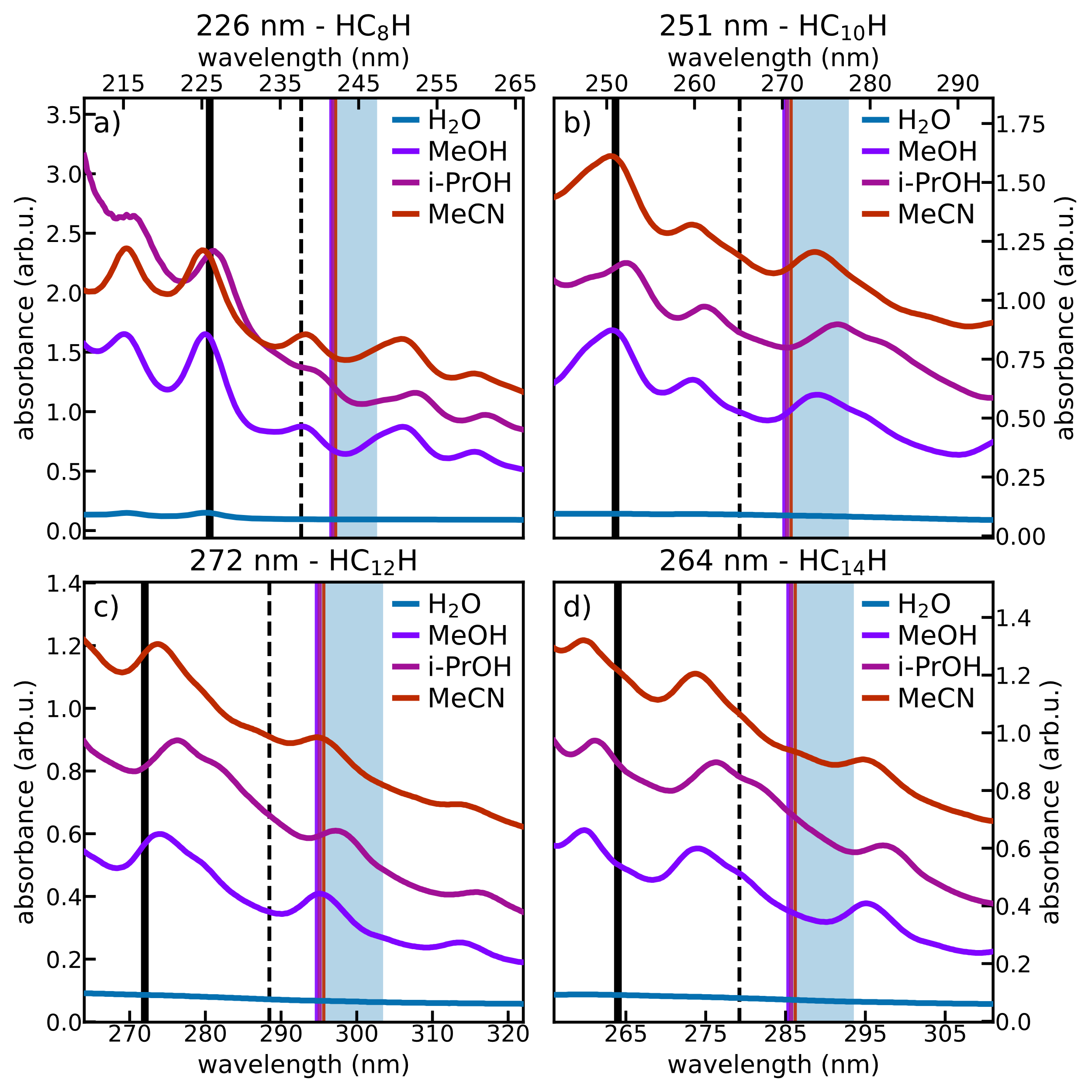


**Figure S8 UV Raman excitation and Raman backscatter photon wavelengths compare to UV-Vis absorption spectra of polyynes mixtures in different solvents.** UV-Vis absorption spectra of water (H_2_O), methanol (MeOH), isopropanol (i-PrOH), and acetonitrile (MeCN) after 15 minutes of ablations of a graphite target in the same conditions of *in situ* experiments. We diluted the mixtures 10 times with pure solvents to reduce the absorbance and avoid instrument saturation. The thick solid black lines represent the Raman excitation at 226 nm (panel a), 251 nm (panel b), 272 nm (panel c), and 264 nm (panel d). The dashed black lines report the wavelength of the Raman photons of the α mode of HC_8_H (panel a), HC_10_H (panel b), HC_12_H (panel c), and HC_14_H (panel d). The violet boxes represent the wavelength range of the OH stretching of water excited at the different Raman excitation wavelengths. The colored lines display the wavelength of the Raman photons of the CH stretching mode of MeOH, i-PrOH, and MeCN excited at the different Raman excitation wavelengths.

Given the self-absorption (SA) issue encountered in UVRR spectra, we established a method to correct our data from SA. In a first approximation, the area of the solvent Raman peaks ($A_{s}(t)$, where $t$ is the ablation time) should remain constant during the measurements ($A_{s}\left( t \right)=A_{s}(0)$). Indeed, if the focal conditions and the power deposited are not changing, we expect the same integrated solvent signal at any ablation time. Thus, these Raman features are good quantifiers for the mixture’s SA, and we choose them as internal references to correct *in situ* UVRR data from SA. In particular, we select the OH stretching band for water and CH stretching modes at around 3000 cm^-1^ for organic solvents. Even though we could use the CN stretching mode from spectra of acetonitrile ablations, we employed CH ones to keep internal coherence among the organic solvents, helping the comparison and avoiding any under- or over-estimation compared to the other solvents.

In such a way, we can correct the integrated polyynes’ signal ($A_{p}(t)$) from the mixture’s SA and precisely evaluate its real dynamics ($A_{p}^{'}(t)$) as a function of the ablation time ($t$) with the following equation:

$$A_{p}^{'}\left( t \right)=A_{p}(t)\frac{A_{s}(0)}{A_{s}(t)}$$

Beyond the current model, we could gain more precise results by using analytical expressions to calculate $A_{p}^{'}\left( t \right)$, like the ones derived in the work of Hong and Asher.[1] These equations require several relevant physical quantities, like the resonance Raman cross section of H-capped polyynes and byproducts and their molar extinction coefficients, which are not available in the literature and are hardly measurable for these compounds. Moreover, they involve a precise knowledge of experimental parameters, like the area of the synchrotron beam in the focal point and the optical path length in the mixture, which cannot be estimated in our setup with the necessary accuracy. Thus, these fine models are practically unusable in complex environments like our *in situ* measurement scheme. This discussion gives more relevance to our empirical model that can easily be adapted to similar experiments. Indeed, we employed this model in a previous work concerning *in situ* surface-enhanced Raman scattering measurements during ablation [2].

# **Conversion model: from integrated UVRR signal to polyynes’ concentration**

We developed a conversion model to transform UVRR integrated signals associated with the α modes of polyynes, obtained through *in situ* UVRR measurements, to the corresponding polyynes’ concentrations. This model serves as a pivotal aspect of our analysis as it establishes a direct link to the production of polyynes.


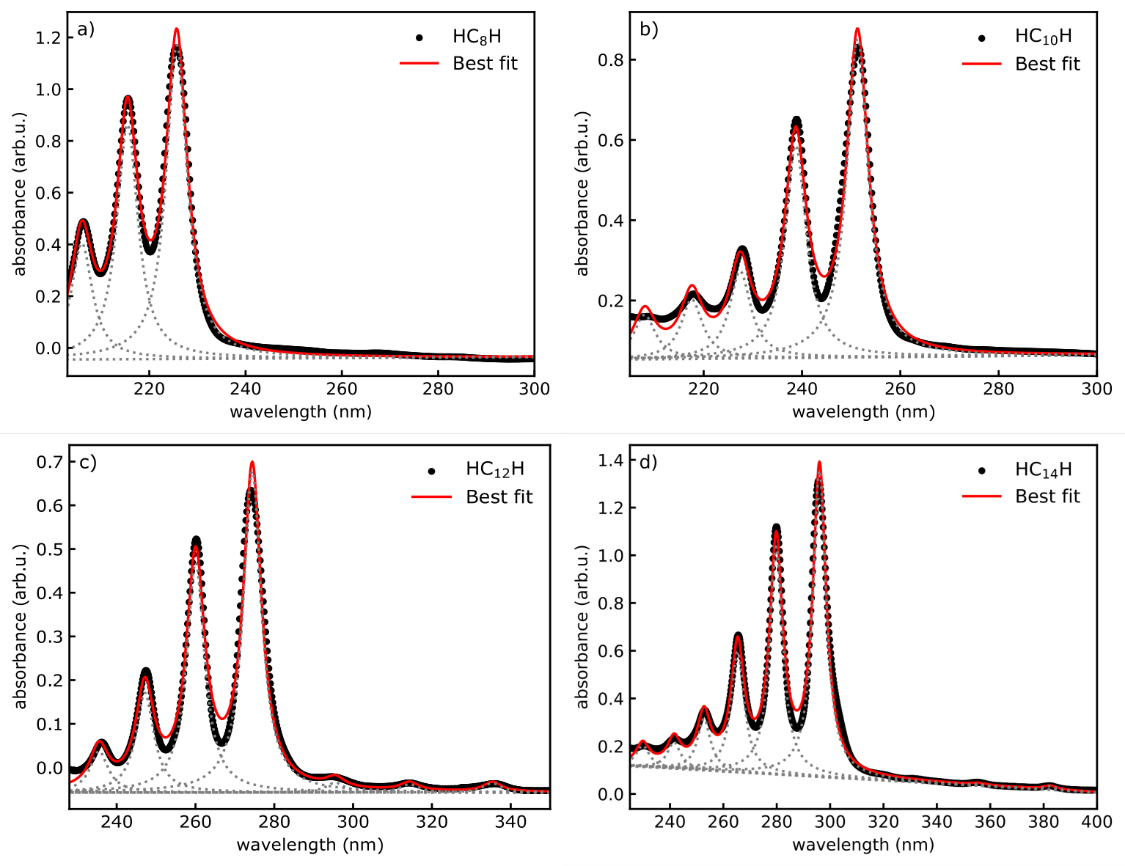


**Figure S9** **UV-Vis spectra of H-capped polyynes.** Experimental UV-Vis absorption spectra of size-selected hydrogen-capped polyynes (black dots), namely a) HC_8_H, b) HC_10_H, c) HC_12_H, and d) HC_14_H. Red solid lines represent best fit functions composed by multi-Lorentzian curves reported as gray dotted lines.

In the initial phase, we collected size-selected samples of HC_8_H, HC_10_H, HC_12_H, and HC_14_H from a high-performance liquid chromatography (HPLC) system using the method described in our previous works.[3,4] These chains are dissolved in acetonitrile/water solutions with different proportions (see the caption of Table S3), corresponding to the mobile phase of our HPLC system. Thus, we conducted UV-Vis absorption measurements on these samples, illustrated in Figure S9. Using these measurements, we employed a multi-Lorentzian fit function, adjusted with a linear baseline, to determine the absorbance of the most prominent peak, corresponding to the 0‒0 vibronic transition of each chain. This information allowed us to calculate the concentration of each polyyne, employing Lambert-Beer’s law and referring to the extinction molar coefficients outlined in Ref. [5]. The concentration of each size-selected polyyne is reported in Table S3.

| **Polyyne** | **Concentration  [10^-6^ mol/L]** | **Excitation wavelength [nm]** | **Power on the sample [μW]** | **Aperture slits [μm]** | **Acquisition time [s]** | **CN stretching mode area (pristine solution)** |
| --- | --- | --- | --- | --- | --- | --- |
| HC_8_H | 6.8 ± 0.03 | 226 | 10.7 | 50 | 10 | 36114 ± 51 |
| HC_10_H | 3.5 ± 0.03 | 251 | 14.3 | 50 | 10 | 21755 ± 22 |
| HC_12_H | 2.5 ± 0.02 | 272 | 9.4 | 30 | 10 | 1381 ± 2 |
| HC_14_H | 4.3 ± 0.02 | 264 | 9.2 | 30 | 10 | 2649 ± 4 |

**Table S3** Sample concentrations and UVRR parameters employed to establish the conversion factor for each polyyne. CN stretching mode’s UVRR integrated signal is extracted from spectra of pristine solutions. Since the solutions are a mixture of acetonitrile and water, we multiplied the area by ≈1.45 (275/175 MeCN/H_2_O), ≈1.29 (310/90 MeCN/H_2_O), ≈1.11 (360/40 MeCN/H_2_O), and ≈1.06 (372/28 MeCN/H_2_O) for HC_8_H, HC_10_H, HC_12_H, and HC_14_H, respectively. The multiplied area is reported in the last column.


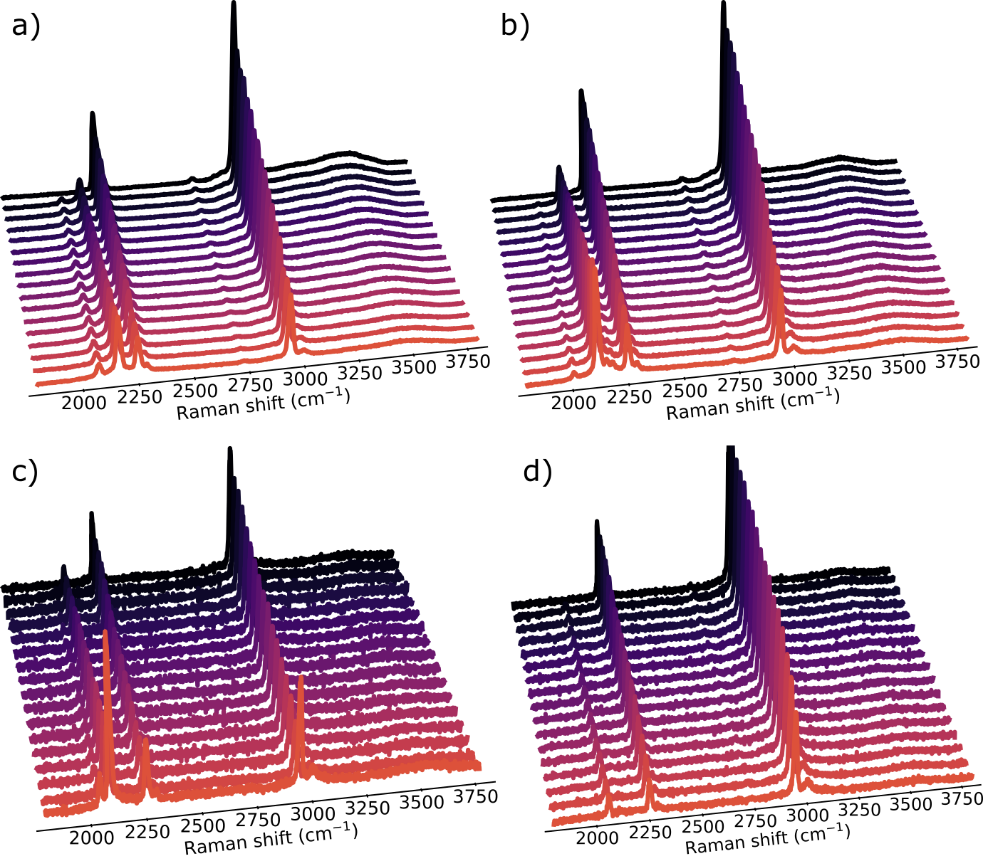


**Figure S10 UVRR spectra of H-capped polyynes at different dilutions.** UV resonance Raman spectra of size-selected hydrogen capped polyynes, namely a) HC_8_H, b) HC_10_H, c) HC_12_H, and d) HC_14_H at increased dilution going from orange (maximum concentration) to purple to black (pure solvent).

Following this approach, we systematically obtained UVRR spectra exciting each polyyne at its 0‒0 vibronic transition, namely 226, 251, and 272 nm for HC_8_H, HC_10_H, and HC_12_H. Due to the limitation of the explorable excitation range related to the technical characteristics of the IUVS beamline discussed in the main text, we employed the 0‒2 vibronic transition at 264 nm to obtain UVRR spectra of HC_14_H. We collected these spectra using the same parameters of *in situ* UVRR measurements, even if the synchrotron-based radiation power and the collection efficiency varied between these two sets (*i.e.*, *in situ* UVRR measurements and UVRR spectra in Figure S10) of experiments, as listed in Table S3. We measured the pristine polyynes’ solutions and collected UVRR spectra after gradually diluting each solution by incrementally adding 20 or 40 μL of a pristine acetonitrile/water solution, maintaining the same proportions as those in the samples containing polyynes. The UVRR spectra of the pristine and diluted solutions for each chain and the reference acetonitrile/water mixtures are shown in Figure S10. We employed a multi-Lorentzian fit function coupled with linear baseline correction to evaluate the UVRR integrated signals of polyynes’ α mode in Figure S10, along with that of solvent (MeCN and water) characteristic signals. The values of the corresponding areas are reported in Figure S11a, c, e, and g. To mitigate the distortion introduced by polyynes’ self-absorption (SA), similarly to what was discussed in the main text, we used the CN stretching mode of MeCN as an internal reference.


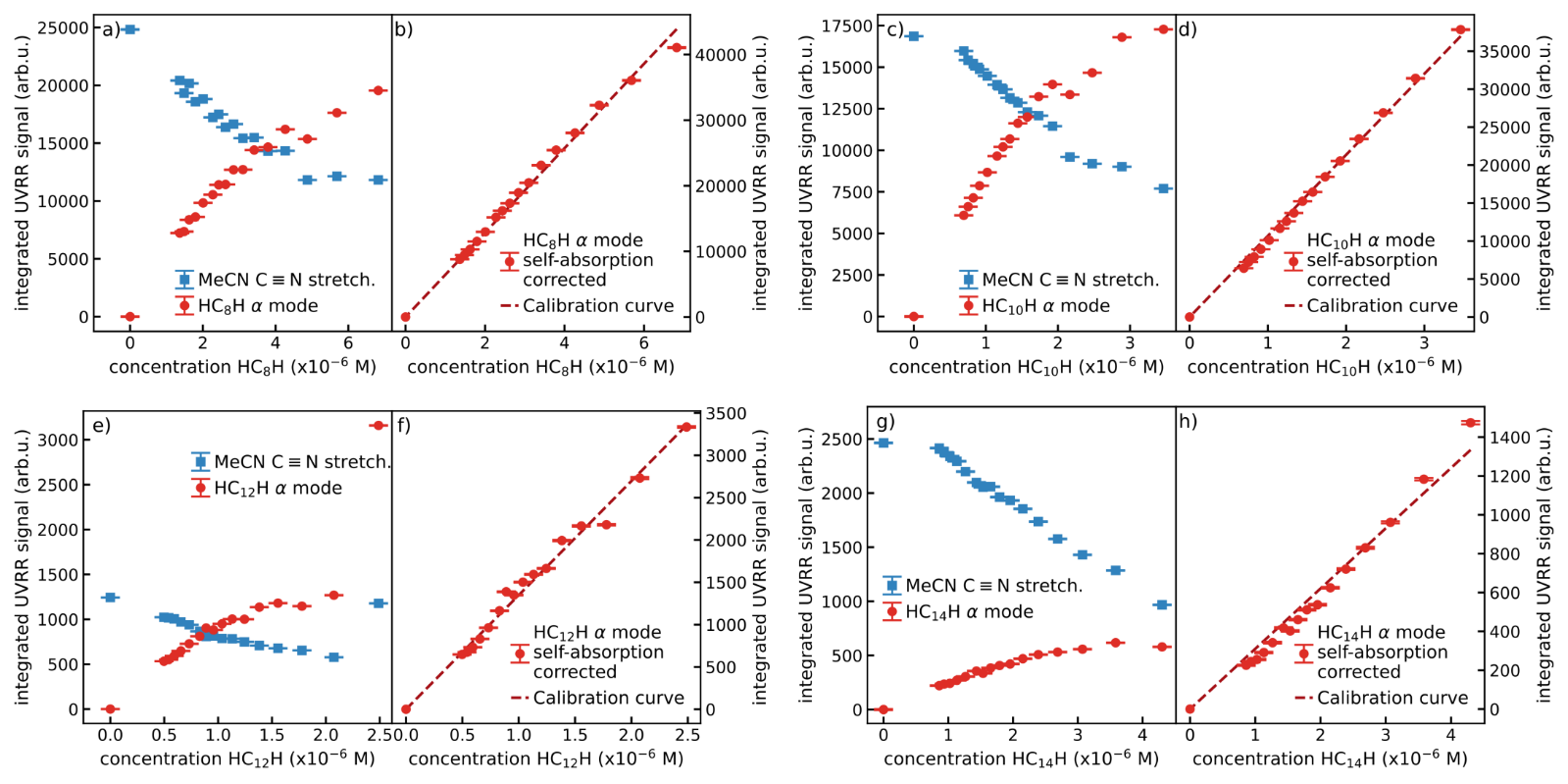
**Figure S11 Polyynes and solvents UVRR integrated signal vs. concentration, uncorrected and corrected from self-absorption.** a) UVRR integrated signal of HC_8_H’s α mode (red dots) and MeCN CN stretching mode (blue squares) excited at 226 nm as a function of HC_8_H’s concentration. b) UVRR integrated signal of HC_8_H’s α mode corrected from self-absorption (red dots) as a function of its concentration. The calibration curve is reported (dark red dashed line). c) UVRR integrated signal of HC_10_H’s α mode (red dots) and MeCN CN stretching mode (blue squares) excited at 251 nm as a function of HC_10_H’s concentration. d) UVRR integrated signal of HC_10_H’s α mode corrected from self-absorption (red dots) as a function of its concentration. The calibration curve is reported (dark red dashed line). e) UVRR integrated signal of HC_12_H’s α mode (red dots) and MeCN CN stretching mode (blue squares) excited at 272 nm as a function of HC_12_H’s concentration. f) UVRR integrated signal of HC_12_H’s α mode corrected from self-absorption (red dots) as a function of its concentration. The calibration curve is reported (dark red dashed line). g) UVRR integrated signal of HC_14_H’s α mode (red dots) and MeCN CN stretching mode (blue squares) excited at 264 nm as a function of HC_14_H’s concentration. h) UVRR integrated signal of HC_14_H’s α mode corrected from self-absorption (red dots) as a function of its concentration. The calibration curve is reported (dark red dashed line).

Consequently, the corrected UVRR area of the α mode of each polyyne ($A_{p}^{'}(c)$) at each concentration ($c$) is calculated using a modified version of Eq. 1 in the main text, where we substituted the ablation time $t$ with the concentration $c$

$$A_{p}^{'}\left( c \right)=A_{p}\left( c \right)\frac{A_{s}\left( 0 \right)}{A_{s}\left( c \right)}$$

Here, $A_{p}(c)$ is the UVRR area of the α mode at polyyne’s concentration $c$, $A_{s}(0)$ and $A_{s}(c)$ are the UVRR integrated signal of the reference solvent’s Raman band, the CN stretching mode of MeCN, in the pristine acetonitrile/water solution ($c=0$) and at polyyne’s concentration $c$. The SA-corrected data are reported in Figure S11b, d, f, and h.

Polyynes-corrected UVRR integrated signals are linearly proportional to their concentration. Using a linear fitting function and forcing the intercept to zero (no polyynes means zero UVRR area), it is possible to find a conversion factor ($k_{UVRR\to c}(p,\lambda)$) to extract the concentration of each polyyne ($p$) at specific Raman excitation wavelengths ($\lambda$). This factor is further adjusted considering all the differences between the *in situ* UVRR data (see Table S1) and this series of spectra (see Table S3). In particular, we multiplied the conversion factor by the ratio between the CN stretching mode’s areas of the two experiments, *i.e.*, *in situ* over *ex situ*. In this way, we will achieve the concentration of polyynes during ablation starting from *in situ* UVRR data. The conversion factors are 4.8 ± 0.05 ∙ 10^9^ (mol/L)^-1^ for HC_8_H, 6.9 ± 0.06 ∙ 10^9^ (mol/L)^-1^ for HC_10_H, 3.4 ± 0.05 ∙ 10^10^ (mol/L)^-1^ for HC_12_H, and 5.5 ± 0.1 ∙ 10^9^ (mol/L)^-1^ for HC_14_H. The errors here reported derive from the fitting procedures used to extract the integrated UVRR signals in *in situ* UVRR, pristine, and diluted spectra and to calculate the conversion factor from the linear calibration curve in Figure S11.

# **Linear production rates and saturated growth regime**


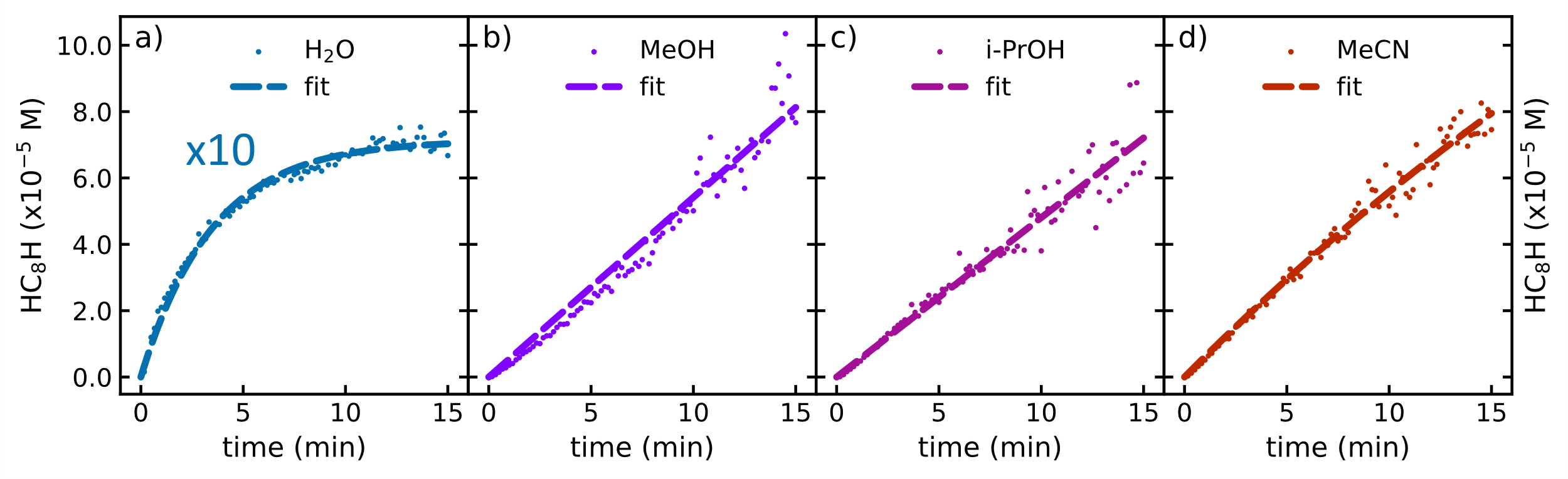

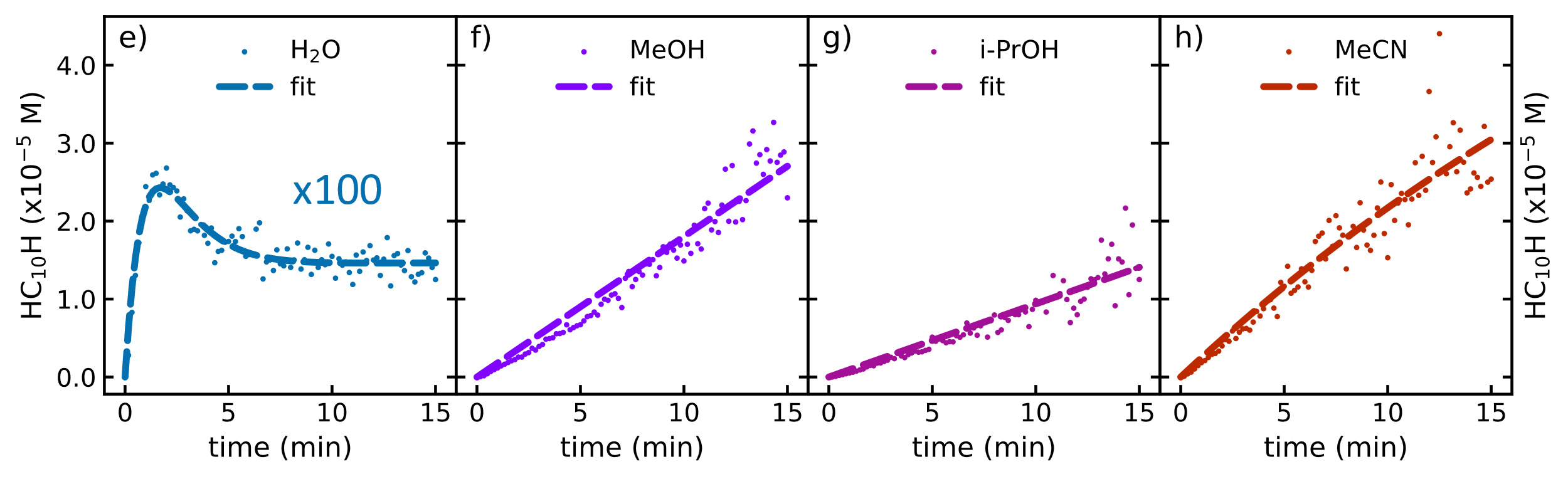

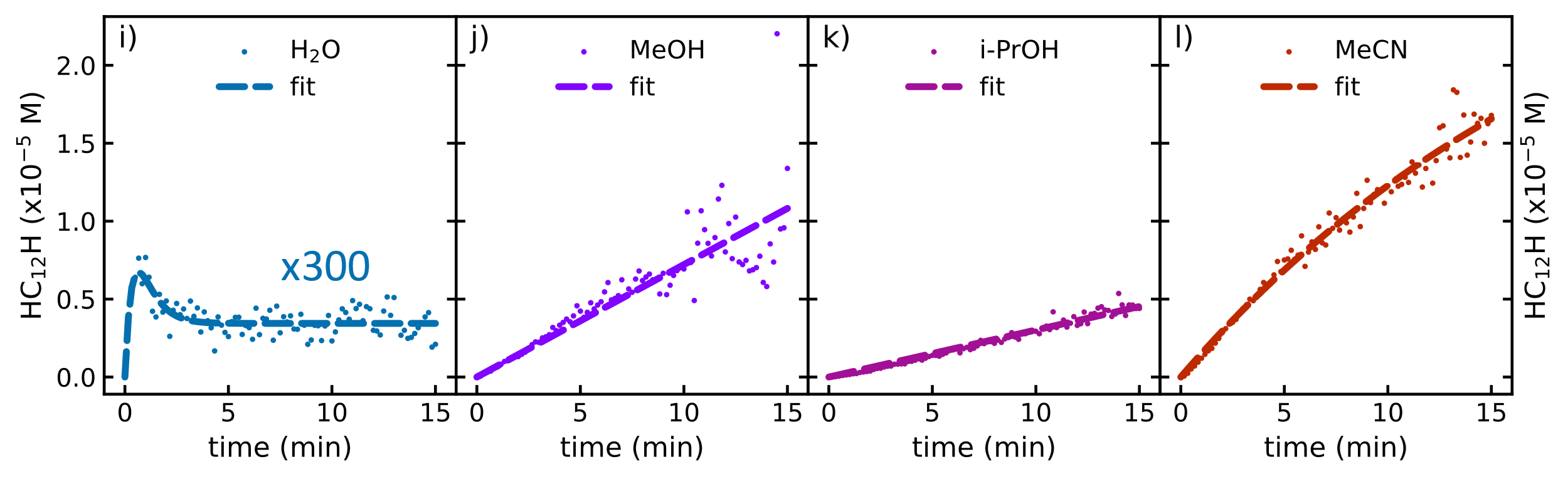


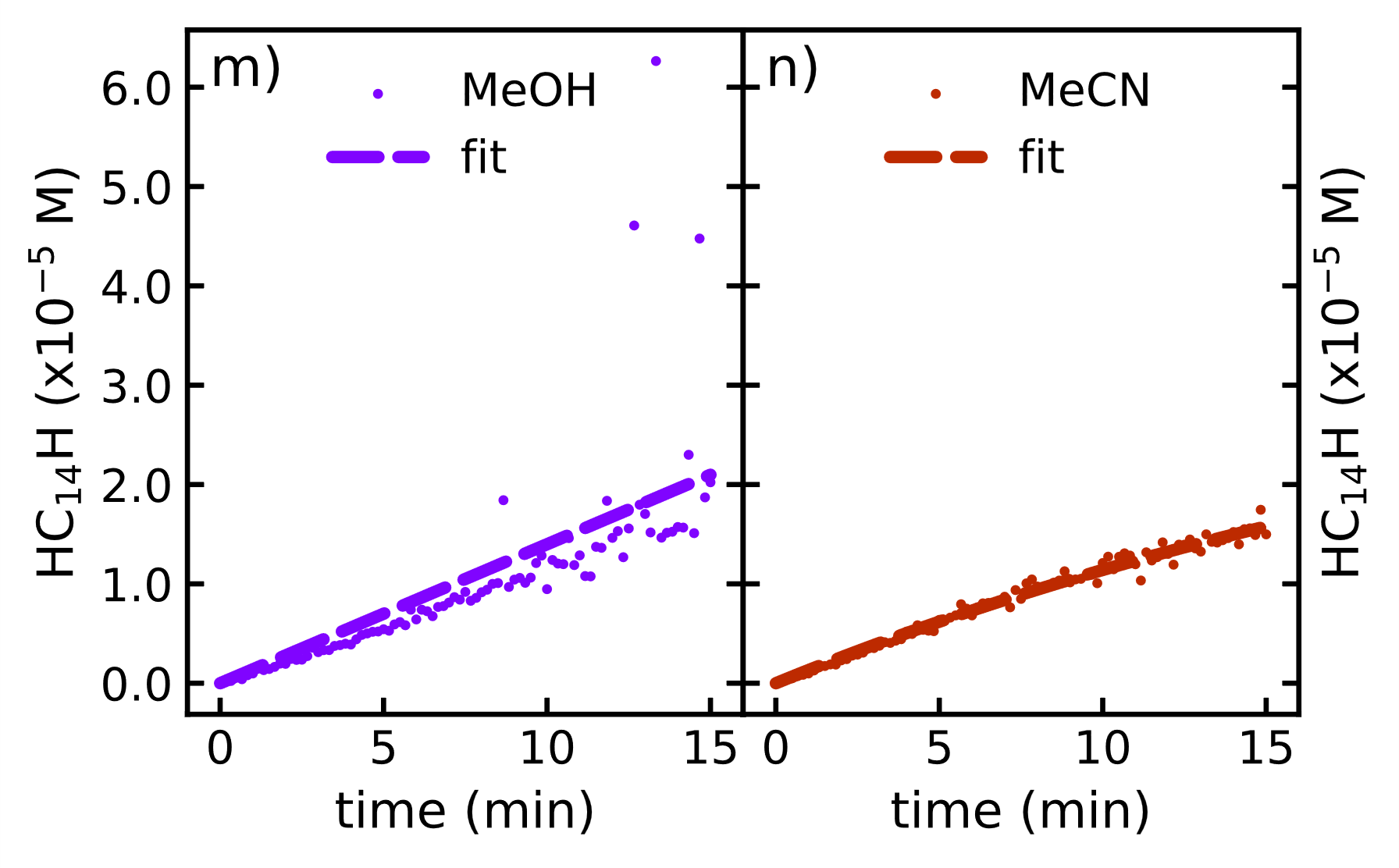


**Figure S12 Evolution and modeling of polyynes concentration during PLAL.** Evolution (colored circles and error bars) and fit (colored dashed lines) result of the SA-corrected concentration of size-selected H-capped polyynes during PLAL in different solvents as a function of the ablation time (refer to Figure 3 for the experimental data). SA-corrected concentration behavior and fit function of HC_8_H extracted from *in situ* UVRR data collected at 226 nm in a) water (modeled with Eq. 3 from the main text), b) methanol (Eq. 5), c) isopropanol (Eq. 5), and d) acetonitrile (Eq. 3). SA-corrected concentration behavior and fit function of HC_10_H extracted from *in situ* UVRR data collected at 251 nm in e) water (Eq. 4), f) methanol (Eq. 5), g) isopropanol (Eq. 5), and h) acetonitrile (Eq. 3). SA-corrected concentration behavior and fit function of HC_12_H extracted from *in situ* UVRR data collected at 272 nm in i) water (Eq. 4), j) methanol (Eq. 5), k) isopropanol (Eq. 5), and l) acetonitrile (Eq. 3). SA-corrected concentration behavior and fit function of HC_14_H extracted from *in situ* UVRR data collected at 264 nm in m) methanol (Eq. 5) and n) acetonitrile (Eq. 3).

|  | HC_8_H [∙10^-6^ M/min] | HC_10_H [∙10^-6^ M/min] | HC_12_H [∙10^-6^ M/min] | HC_14_H [∙10^-6^ M/min] |
| --- | --- | --- | --- | --- |
| H_2_O | 2.04 ± 0.05 | ‒ | ‒ | ‒ |
| MeOH | 5.42 ± 0.07 | 1.80 ± 0.03 | 0.72 ± 0.02 | 1.40 ± 0.08 |
| i-PrOH | 4.81 ± 0.06 | 0.94 ± 0.02 | 0.302 ± 0.003 | ‒ |
| MeCN | 6.22 ± 1.95 | 2.49 ± 1.58 | 1.54 ± 0.26 | 1.35 ± 0.24 |

**Table S4** Production rates in M/min (M is molarity, mol/L) extracted from the fitting procedures of the evolution of the corresponding concentration curves (see Figure S12). See the main text for further details. The fit errors are reported as well.

|  |  | HC_8_H | HC_10_H | HC_12_H | HC_14_H |
| --- | --- | --- | --- | --- | --- |
| H_2_O | Saturation concentration [∙10^-6^ M] | 7.1 ± 0.1 | 0.15 ± 0.02 | 0.011 ± 0.005 | ‒ |
|  | Saturation time [min] | 10.5 ± 0.2 | 0.44 (≈26 s) ± 0.44 | 0.14 (≈8 s) ± 0.06 | ‒ |
| MeCN | Saturation concentration [∙10^-4^ M] | 2.8 ± 0.6 | 0.9 ± 0.4 | 0.33 ± 0.03 | 0.39 ± 0.04 |
|  | Saturation time [min] | 137 ± 32 | 106 ± 51 | 64 ± 8 | 87 ± 12 |

**Table S5** Saturation concentration (in M) and saturation time to reach 95 % of the saturation concentration (in min) extracted from the fitting procedures of the evolution of the concentration curves in water and acetonitrile (see Figure S12). The fit errors are reported as well.

| HC_16_H | HC_18_H | HC_20_H | HC_22_H | HC_24_H | HC_26_H | HC_28_H | HC_30_H |
| --- | --- | --- | --- | --- | --- | --- | --- |
| 3.99 ∙ 10^-6^ | 1.37 ∙ 10^-6^ | 4.74 ∙ 10^-7^ | 1.63 ∙ 10^-7^ | 5.63 ∙ 10^-8^ | 1.94 ∙ 10^-8^ | 6.69 ∙ 10^-9^ | 2.31 ∙ 10^-9^ |

**Table S6** Saturation concentrations (in M) extracted from fitting the data in Figure 5a in the main text (or in Table S5).

# **References**

[1] Hong Z, Asher SA. Dependence of Raman and Resonance Raman Intensities on Sample Self-Absorption. Appl Spectrosc 2015;69:75–83. https://doi.org/10.1366/14-07531.

[2] Marabotti P, Peggiani S, Facibeni A, Serafini P, Milani A, Russo V, et al. In situ surface-enhanced Raman spectroscopy to investigate polyyne formation during pulsed laser ablation in liquid. Carbon 2022;189:219–29. https://doi.org/10.1016/j.carbon.2021.12.060.

[3] Marabotti P, Tommasini M, Castiglioni C, Serafini P, Peggiani S, Tortora M, et al. Electron-phonon coupling and vibrational properties of size-selected linear carbon chains by resonance Raman scattering. Nat Commun 2022;13:5052. https://doi.org/10.1038/s41467-022-32801-3.

[4] Marabotti P, Tommasini M, Castiglioni C, Peggiani S, Serafini P, Rossi B, et al. Synchrotron-based UV resonance Raman spectroscopy probes size confinement, termination effects, and anharmonicity of carbon atomic wires. Carbon 2024;216:118503. https://doi.org/10.1016/j.carbon.2023.118503.

[5] Eastmond R, Johnson TR, Walton DRM. Silylation as a protective method for terminal alkynes in oxidative couplings. Tetrahedron 1972;28:4601–16. https://doi.org/10.1016/0040-4020(72)80041-3.
